# Habitat fragmentation and vegetation structure impact gastrointestinal parasites of small mammalian hosts in Madagascar

**DOI:** 10.1002/ece3.7526

**Published:** 2021-05-01

**Authors:** Frederik Kiene, Bertrand Andriatsitohaina, Malcolm S. Ramsay, Romule Rakotondravony, Christina Strube, Ute Radespiel

**Affiliations:** ^1^ Institute of Zoology University of Veterinary Medicine Hannover Hanover Germany; ^2^ Centre for Infection Medicine Institute for Parasitology University of Veterinary Medicine Hannover Hanover Germany; ^3^ Ecole Doctorale Ecosystèmes Naturels (EDEN) University of Mahajanga Mahajanga Madagascar; ^4^ Department of Anthropology University of Toronto Toronto Canada; ^5^ Faculté des Sciences, de Technologies et de l’Environnement University of Mahajanga Mahajanga Madagascar

**Keywords:** edge effects, *Eliurus*, habitat degradation, life cycle, *Microcebus*, *Rattus*

## Abstract

Deleterious effects of habitat loss and fragmentation on biodiversity have been demonstrated in numerous taxa. Although parasites represent a large part of worldwide biodiversity, they are mostly neglected in this context. We investigated the effects of various anthropogenic environmental changes on gastrointestinal parasite infections in four small mammal hosts inhabiting two landscapes of fragmented dry forest in northwestern Madagascar. Coproscopical examinations were performed on 1,418 fecal samples from 903 individuals of two mouse lemur species, *Microcebus murinus* (*n* = 199) and *M. ravelobensis* (*n* = 421), and two rodent species, the native *Eliurus myoxinus* (*n* = 102) and the invasive *Rattus rattus* (*n* = 181). Overall, sixteen parasite morphotypes were detected and significant prevalence differences between host species regarding the most common five parasites may be explained by parasite–host specificity or host behavior, diet, and socioecology. Ten host‐ and habitat‐related ecological variables were evaluated by generalized linear mixed modeling for significant impacts on the prevalence of the most abundant gastrointestinal parasites and on gastrointestinal parasite species richness (GPSR). Forest maturation affected homoxenous parasites (direct life cycle) by increasing *Lemuricola*, but decreasing Enterobiinae gen. sp. prevalence, while habitat fragmentation and vegetation clearance negatively affected the prevalence of parasites with heterogenic environment (i.e., *Strongyloides* spp.) or heteroxenous (indirect cycle with intermediate host) cycles, and consequently reduced GPSR. Forest edges and forest degradation likely change abiotic conditions which may reduce habitat suitability for soil‐transmitted helminths or required intermediate hosts. The fragility of complex parasite life cycles suggests understudied and potentially severe effects of decreasing habitat quality by fragmentation and degradation on hidden ecological networks that involve parasites. Since parasites can provide indispensable ecological services and ensure stability of ecosystems by modulating animal population dynamics and nutrient pathways, our study underlines the importance of habitat quality and integrity as key aspects of conservation.

## Graphical abstract

This study reveals negative effects of habitat fragmentation, habitat degradation, and forest edges on prevalences of intestinal parasites with complex life cycles that were found in four small, arboreal mammal species in fragmented dry forest landscapes in northwestern Madagascar. The effects were explained as consequences of unfavorable abiotic conditions constraining free‐living parasite stages or arthropod intermediate hosts. As parasites fulfill essential functions in ecosystems, forest fragmentation threatens not only parasite biodiversity but also ecosystem integrity.

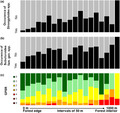

## 1 INTRODUCTION

The natural world is highly impacted by human activities of various kinds (Jha & Bawa, 2006). Most importantly, land conversion leads to overall size reduction and an increasing degree of fragmentation of the remaining natural habitats, threatening biodiversity. The most diverse terrestrial habitats are tropical forests (Cincotta et al., 2000; Myers et al., 2000). They cover less than 10% of the world's land area, but harbor more than 60% of all terrestrial species (Gardner et al., 2009; Harper et al., 2007; Lovejoy, 1997; Mayaux et al., 2005). Negative effects of tropical forest fragmentation on species diversity have been studied in the context of species–area relationships in many taxonomic groups (Fahrig, 2003). The dependence of species abundance on habitat size was for example revealed in mammals (Andren, 1994; Andriatsitohaina et al., 2020; Crooks et al., 2011, 2017; Klass et al., 2020; Steffens, 2017), birds (Andren, 1994; Watson et al., 2005), reptiles (Hager, 1998; Mac Nally & Brown, 2001), amphibians (Cushman, 2006; Hager, 1998; Kolozsvary & Swihart, 1999), invertebrates (Didham, 1997), fungi (Vannette et al., 2016), and plants (Raghubanshi & Tripathi, 2009; Tabarelli et al., 1999). Parasites have been largely neglected in this context, although they account for more than 40% of the species on our planet (Bordes & Morand, 2009; Dobson et al., 2008; Gómez & Nichols, 2013). The species diversity of parasitic helminths alone is estimated to be 50% higher compared with the diversity of vertebrate species serving as their hosts (Poulin & Morand, 2000). Parasites are essential components of ecosystems and act as regulators of host population dynamics and community structure (Dunne et al., 2013; Lafferty et al., 2006, 2007; Marcogliese, 2004; Mouritsen & Poulin, 2005; Thomas et al., 1999). Whereas ecological impacts of habitat fragmentation on ectoparasites (e.g., mites, ticks) can be expected given their more direct exposure to external abiotic conditions (Bush et al., 2013; Carbayo et al., 2019; Kiene et al., 2020), the response of endoparasites to habitat fragmentation is less intuitive. However, depending on their life cycle, endoparasites can be exposed to direct environmental influences as free‐living stages or in intermediate hosts (Simões et al., 2016). Only few studies so far compared gastrointestinal parasite prevalences in different vertebrate hosts between continuous habitats and habitat fragments. Whereas some found higher prevalences in hosts from disturbed and fragmented areas (Froeschke et al., 2013; Gillespie & Chapman, 2006, 2008; Gillespie et al., 2005; Klaus et al., 2018; Trejo‐Macías et al., 2007), others showed that hosts from pristine, continuous habitats exhibited higher infection rates (Gay et al., 2014; Martínez‐Mota et al., 2018; Resasco et al., 2019; Taylor & Merriam, 1996; Vandergast & Roderick, 2003). The reasons for those contradictory results remain unclear (Bordes et al., 2015).

The fragmented forests of Madagascar represent a highly suitable model region to investigate impacts of habitat fragmentation and degradation on parasites. The islands’ forests are under particular pressure (Harper et al., 2007), since the human population of Madagascar has grown from around 4 million people in 1950 to almost 27 million people in 2019 (United Nations, 2019). By 2014, natural forest cover decreased to 56% of its size in 1953 (Vieilledent et al., 2018). In parallel, human impact on the remaining forests and the potential for ecological edge effects increased considerably, since 46% of the remaining forest areas are located closer than 100 m to a forest edge (Vieilledent et al., 2018). Malagasy forest ecosystems harbor an extraordinary and unique species richness and are considered a worldwide hotspot for biodiversity (Goodman & Benstead, 2005; Raik, 2011). A few studies investigated the effects of forest fragmentation on ectoparasite infections, however with contrasting results. For example, Ehlers et al. (2019) could not detect habitat effects on ectoparasites of various mammalian hosts and domestic chicken. In contrast, Junge et al. (2011) suggested a higher susceptibility of *Indri indri* for ectoparasites in degraded habitats by focusing on the general health status of this large lemur species. Conversely, Kiene et al. (2020) found significantly lower ectoparasite infestation rates in small mammalian hosts from smaller forest fragments and in proximity to the forest edge. While almost all ectoparasite types were affected, effects were particularly evident in temporary ectoparasites such as ticks and chigger mites. The authors argued that ectoparasite survival and reproduction of temporary parasite stages in edge environments are most likely reduced by unfavorable abiotic environmental conditions at the forest edge compared with the forest interior (Kiene et al., 2020).

Some studies investigated gastrointestinal parasite infections of Malagasy vertebrates in relation to habitat disturbance and degradation and again with different results: Rakotoniaina et al. (2016) found no effect of habitat degradation on gastrointestinal parasites in gray mouse lemurs (*Microcebus murinus*), while the study of Raharivololona and Ganzhorn (2009) suggested that effects differ between parasite species, but the investigation was based on very small sample size. Studies on homoxenous pinworms of larger brown lemurs (*Eulemur* spp.), however, found higher prevalences in animals inhabiting secondary forests or previously logged habitats (Schwitzer et al., 2010; Winter et al., 2020), but the studies are lacking information on heteroxenous parasites. To our knowledge, no study has so far investigated the complex effects of habitat fragmentation on gastrointestinal parasites with different life cycle characteristics.

Our study evaluates the effects of habitat fragmentation and degradation on gastrointestinal parasite prevalence and species composition in three endemic and one invasive small mammalian host species living in fragmented dry deciduous forest landscapes in northwestern Madagascar. All four host species are relatively small (endemic primates: *Microcebus murinus:* ~54 g, *M. ravelobensis:* ~56 g; endemic rodent: *Eliurus myoxinus:* ~66 g; invasive rodent: *Rattus rattus:* ~100 g), are known to occur in larger numbers even in forest fragments, and live in partial sympatry in the study region (Andriatsitohaina et al., 2020). All of them are nocturnal solitary foragers, exhibit a more or less arboreal lifestyle, and spend the day in protected sleeping sites. The four studied species, however, also differ in important aspects of their biology: the two *Microcebus* species and *R. rattus* are living in groups and feed on an omnivorous diet (Clark, 1982; Radespiel, 2000; Radespiel et al., 2006; Shiels & Pitt, 2014; Thorén et al., 2011; Weidt et al., 2004), while the western tuft‐tailed rat has a solitary lifestyle and is categorized as frugivorous (Goodman, 2016; Randrianjafy et al., 2008). The rodents and *M. murinus* mainly use tree holes and dens for sleeping, each of them for longer stretches of time (Goodman, 2016; Münster, 2003; Radespiel et al., 2003), while *M. ravelobensis* is known to employ more open and ephemeral shelters such as self‐built leaf nests and switch between sites more frequently (Radespiel et al., 2003; Thorén et al., 2010).

Based on these differences in host biology, omnivorous host species (mouse lemurs, *R. rattus*) that also feed on insects which may act as arthropod intermediate hosts can be expected to show higher rates of infection and a higher diversity of gastrointestinal parasites than the frugivorous *E. myoxinus*. Moreover, hosts showing long‐term use of sheltered sleeping sites (rodents, *M. murinus*) might exhibit increased infection rates in comparison with hosts which use less sheltered and ephemeral sleeping sites (*M. ravelobensis*). In addition to host species, impacts of host sex and host population density can be anticipated. Male hosts are expected to exhibit higher gastrointestinal parasite prevalences compared with females, since specific male behavior is known to foster parasite infections (Altizer et al., 2003; Klein, 2004; Poirotte & Kappeler, 2019; Zuk & Mckean, 1996). Hosts from habitats with higher population density can be expected to show elevated gastrointestinal parasite infection rates, since increasing social interactions and density‐associated higher parasite contamination of the environment might increase infection risk (Arneberg, 2002). In addition to these host‐related factors, external environmental factors (e.g., forest size, proximity to the forest edge, vegetation structure, and human disturbance) can also be expected to impact infections, since gastrointestinal parasites spend periods of their life as free‐living stages in the environment or rely on arthropod intermediate hosts. Thus, gastrointestinal parasites can be predicted to be negatively impacted by forest edges and habitat degradation, since lower humidity and UV radiation in edge and degraded habitats can be suspected to increase mortality of parasites and availability of arthropod intermediate hosts.

However, as gastrointestinal parasites should be overall more protected from external environmental influences than ectoparasites, we expected a weaker response than in the previous study on ectoparasites (Kiene et al., 2020). Host‐related factors, in contrast, are expected to explain most of the prevalence variation, although their investigation in a nonexperimental setting precludes full clarification of causality.

## 2 MATERIAL AND METHODS

### 2.1 Study regions

**FIGURE 1:**
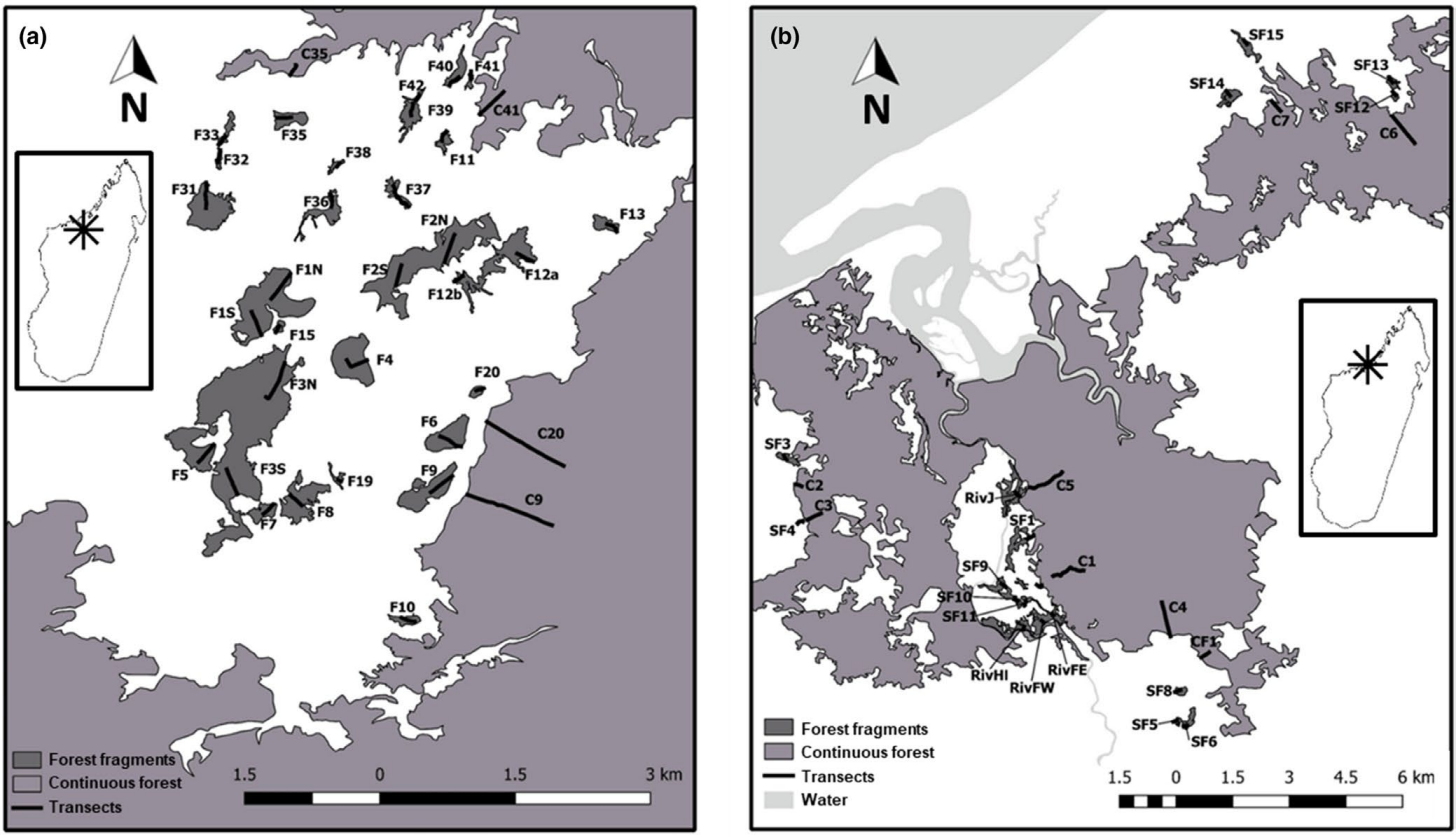
Maps of two studied networks of fragmented dry deciduous forest, one in the western part of the Ankarafantsika National Park (a) and one in the Mariarano region (b). Modified after Andriatsitohaina et al. (2020)

Two fragmented landscapes of dry deciduous forest patches, about 90 km apart from each other, were studied in the dry seasons (May to October) of the years 2017 and 2018. Both are situated within the Boeny region in northwestern Madagascar: The Ankarafantsika National Park (ANK; 16°180S, 46°420E, 75 km southeast of the city of Mahajanga; Figure 1a) and the Mariarano Classified Forest (MAR; 15°240S, 46°440E, 50 km northeast of Mahajanga; Figure 1b). Major differences between the two locations concern elevation, disturbance by human presence, the type of landscape separating forest fragments (= matrix), and the availability of surface water. Situated on a plateau at about 180 m above sea level (a.s.l.), the forest sites in ANK are surrounded by a homogeneous, dry grassland matrix dominated by *Aristida barbicollis* (Steffens & Lehman, 2016). Open water bodies are completely absent and expansion of forest vegetation into the matrix is mostly prevented by cattle herding and recurrent bushfires (Ramsay et al., 2020). The forest patches in MAR are located closer to the Mozambique Channel (4–15 km) at an elevation of 20–90 m a.s.l. and managed by the local municipality Mariarano, which is situated in the center of the area. Here, the fragmented dry deciduous forests are embedded in a rather heterogeneous matrix consisting of rice fields or of savannah‐like grasslands with palm trees (*Bismarckia nobilis*) in varying densities. Ponds, streams, channels for field irrigation, and the Mariarano River provide some humidity throughout the year, and the riverine vegetation maintains a potential connection between some of the forest fragments. In general, a relatively cool dry season from May to October and a hot and humid rainy season from November to April ensure a highly seasonal climate in the entire region.

The software QGIS (QGIS Development Team, 2018, http://qgis.osgeo.org) was used to map the study areas and determine distances and surface measures based on GPS data which was collected by walking along transects and forest edges. Forest edges were identified by following the definition of Steffens (2017). Estimates of larger continuous forests were based on Google Earth Pro satellite footage after completion of the sampling period (Google, 2018. Google Earth Pro, version 7.3.2., http://www. earth.google.com [accessed in December 2018]).

### 2.2 Recording of vegetation data

**FIGURE 2:**
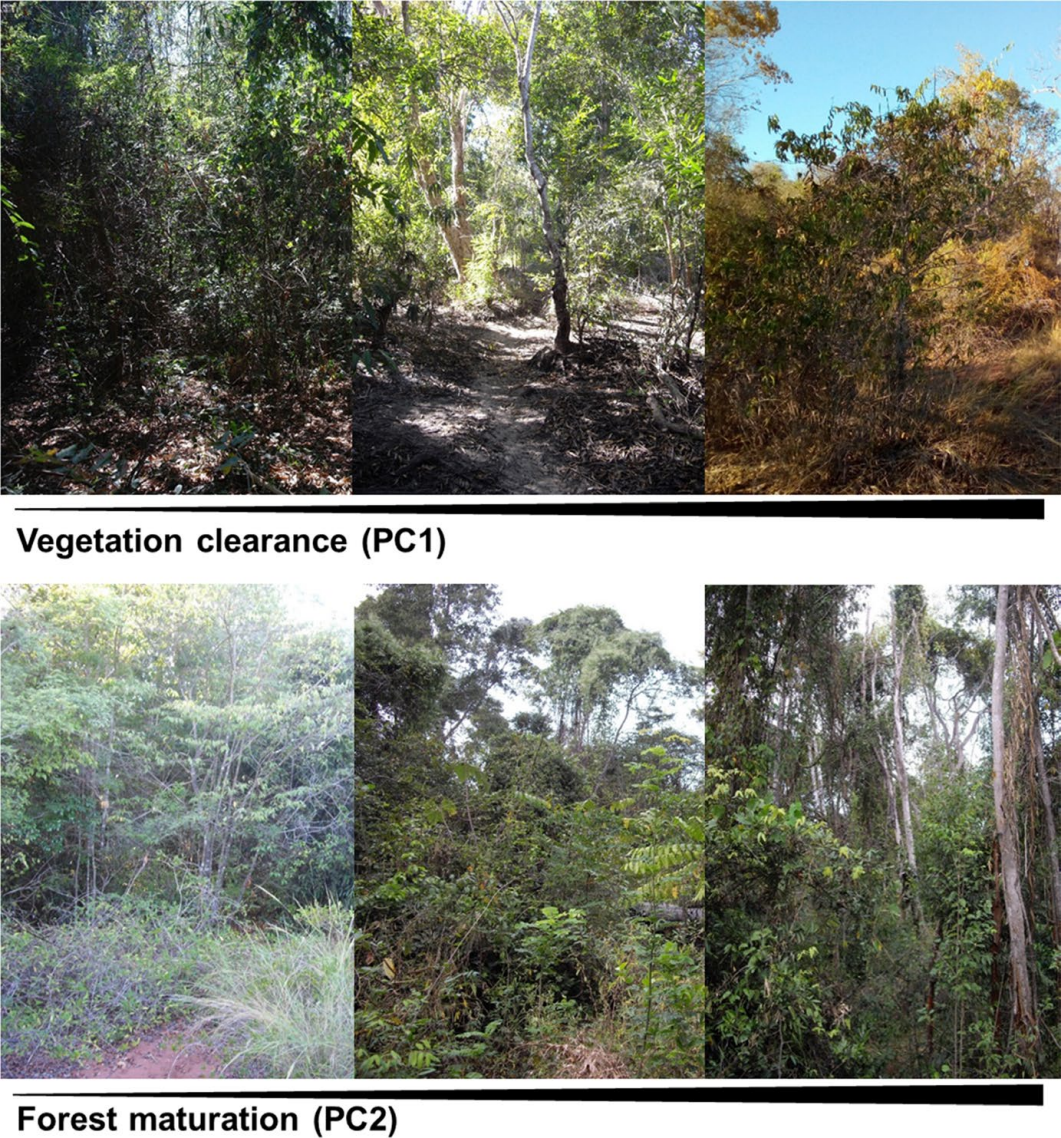
Illustration of varying levels of vegetation clearance (from dense forest vegetation to more open growth interspersed with grassy vegetation, PC1) and forest maturation (from low secondary growth to mature forest with tall old growing trees, PC2). The arrows under the photographs symbolize increasing values of PC1 and PC2

Vegetation density data were collected by counting seedlings (height: 1–100 cm), saplings (height: 101–250 cm), trees (height >250 cm), and lianas (diameter at breast height ≥2.5 cm) within plots of a size of 2 × 10 m according to Malcolm et al. (2016). Plots were installed in pairs, orthogonally directed from the forest transects, at a predefined set of distances to the forest edge (0, 20, 40, 60, 80, 100, 200, 250, 300 m, then every 100 m). Thus, 16 pairs of vegetation plots were evaluated along a 1,000 m transect. In addition, the number of signs of disturbance by human presence per plot (number of cut trees, large holes in the ground as residuals from maciba (*Dioscorea* spp.) root harvesting as well as zebu scats) was recorded. To condense data on vegetation structure and human disturbance, and to include these into the subsequent generalized linear mixed modeling, a principal component analysis (PCA) was performed. For each 100 m segment of a transect, average values of seedling, sapling, tree, liana, cut tree, maciba hole, and zebu scat counts across all vegetation plots were used as data points for the PCA. Resulting principal components (PC) were finally attributed to each host individual captured at trap positions within the respective segment. The PCA was performed using the R‐command “prcomp()”. The principal components (PC1, PC2) with Eigenvalues of >1 and a high explanatory power (PC1: 26.2%, PC2: 18.5%) were selected as predictor variables for the subsequent modeling. Factor loadings were utilized to interpret their effects (correlation coefficients; Table 1; File S1). Lower densities of trees, seedlings and saplings, and higher numbers of cut trees and zebu scat were associated with increasing values of PC1 (Table 1). Consequently, PC1 is primarily associated with changes in general vegetation density. With increasing PC1, the vegetation opens up and forests are more frequently used by zebus, indicating increasing human impact (Figure 2). We consequently interpret PC1 as factor illustrating “vegetation clearance”. In contrast, PC2 is rather connected to changes along a gradient from secondary to primary forest vegetation (Figure 2). It is linked to lower numbers of cut trees, seedlings, saplings and lianas, and higher numbers of maciba holes (Table 1). The decreasing number of cut trees and the reduced understory (less seedlings and saplings) along an increasing PC2 suggest the presence of larger old growing trees indicative of a mature primary forest. Although more maciba holes could indicate higher human impact, they also imply a greater density of maciba plants, which depend on a more pristine ecosystem (Andriamparany et al., 2015). For readability, we consequently interpret PC2 as factor illustrating “forest maturation”.

**TABLE 1:** Factor loadings of principal component (PC) 1 (vegetation clearance) and PC 2 (forest maturation)

	No. of seedlings	No. of saplings	No. of trees	No. of lianas	No. of maciba holes	No. of cut trees	No. of zebu scats
PC1	−0.417	−0.358	−0.553	−0.240	−0.249	0.280	0.441
PC2	−0.415	−0.304	−0.060	−0.208	0.651	−0.515	0.017

### 2.3 Host capture and fecal sampling

Host animals were captured in Sherman live traps (Sherman Traps, Inc.) baited with pieces of banana. Traps were installed on tree branches or in bushes at heights varying between 0.5 and 2.5 m. Pairs of traps were placed every 10 m along transects leading perpendicular from the forest edge to the interior of a continuous forest or to the center of a forest fragment. Transects in forest fragments ranged from 40 to 490 m in length, transects in continuous forests ranged from 150 to 1,000 m. In total, 49 transects (31 in ANK, 18 in MAR; Figure 1) were installed in 40 forest fragments (0.8–114.6 ha), and 12 transects (4 in ANK, 8 in MAR; Figure 1) were installed in four much larger “continuous” forests (3,683–130,390 ha). Traps were set in the late afternoon and checked in the early morning of the following day. Trapping was conducted three times per transect at intervals of about 3 days. The number of animals of a species captured per 100 installed traps was calculated as an approximation of host population density. Handling and sampling were conducted during the morning, and animals were released at their specific capture positions in the evening of the same day. The rodents, particularly susceptible to stress during examinations (Artwohl et al., 2006), were anesthetized with ketamine and xylazine (Ketamine 10%, Medistar, Germany; Xylazine 20 mg/ml, Serumwerk Bernburg, Germany; 80 mg/kg ketamine + 16 mg/kg xylazine for *R. rattus*, 70 mg/kg ketamine + 12 mg/kg xylazine for *E. myoxinus*), injected into the gluteus maximus muscle of the hind limb. All animals were individually marked with coded ear clippings, weighed, and sexed. Morphometric measurements were recorded according to Hafen et al. (1998). Head width and body weight were, separated by species, inserted into the equation of Peig and Green (2009) to compute the scaled mass index (SMI) as an individual approximation for body condition. To be able to compare the SMI of the different species, values were divided by the species median for standardization. Fecal samples (0.04–2.0 g) were taken directly from the anus or collected from the traps, which were cleaned and disinfected prior to installation. Samples were directly preserved in 1.5 ml ethanol (90%–96%) and stored at 4°C.

### 2.4 Parasite identification

Parasite stages were detected in the preserved feces by applying a combined flotation–sedimentation method. Up to three samples per host animal were pooled in a 15‐ml tube and centrifuged at 1,400×*g* for 5 min to discard supernatant ethanol. After weighing the samples, saturated zinc sulfate solution (specific gravidity: 1.3) was added to 15 ml and vigorously mixed. To float the parasite stages, samples were centrifuged at 250×*g* for 10 min. The supernatant was sieved with tap water into a new tube, where parasite stages were sedimented by centrifugation at 450×*g* for 5 min. The sediment was transferred into a counting chamber and parasite eggs, oocysts, and larvae were microscopically classified into morphotypes, based on different references for primate (Cameron, 1930; Chabaud et al., 1965; Rosario Robles et al., 2010; Hugot et al., 1995; Hugot & del Robles, 2011; Irwin & Raharison, 2009; Kalousová et al., 2014; Little, 1966; Radespiel et al., 2015; Raharivololona, 2006, 2009) as well as rodent parasites (Baker, 2006, 2008; Bowman et al., 2004; Dewi et al., 2018; Khalil et al., 2014; Petrzelkova et al., 2006; Sambon, 1924; Smales et al., 2009; Thomas, 1924).

For molecular verification, a minimum of one subsample of each parasite morphotype from each host species was selected for analysis. DNA was isolated from 5 to 50 eggs or larvae per sample with the NucleoSpin©Tissue kit (MACHEREY‐NAGEL). Egg morphotypes with strong shells were homogenized using Precellys^®^ ceramic bead tubes (Bertin Instruments) prior to DNA isolation. The rDNA region spanning the internal transcribed spacer (ITS)1–5.8S–ITS2 sequence (hereafter referred to as ITS sequences) has been shown to be of excellent use for the taxonomic classification of nematodes (Blouin, 2002; Nabavi et al., 2014) and was therefore chosen for this study. Newly generated sequences were deposited in GenBank under accession nos. MW520838–MW520847, MW520852, and MW520853. Details of methods and the results of this molecular taxonomic evaluation are described in File S2 and were used to support parasite morphotype classification down to the family, genus, or species level, whenever possible. Those derived names are used throughout the manuscript.

### 2.5 Data analyses

The gastrointestinal parasite species richness (GPSR), defined as the number of simultaneously present gastrointestinal parasite morphotypes in the feces of an individual host, and presence–absence data of the five most prevalent parasite morphotypes (>5% total prevalence) in 903 individual hosts were used as dependent variables in generalized linear mixed models (GLMMs). The “lme4” package was employed for computation of GLMMs using the software R (R Foundation for Statistical Computing, Vienna, Austria, https://www.R‐project.org) and RStudio (Integrated Development for RStudio, Inc., http://www.rstudio.com). All dependent variables were related to a suite of 10 biologically meaningful predictor variables (4× host‐related, 6× environmental) and one interaction term. Host‐related predictor variables were host species (*M. murinus*, *M. ravelobensis*, *E. myoxinus*, *R. rattus*), host sex (male, female), host population density (number of captured hosts of a species per 100 installed traps), and body condition (SMI divided by the species median value). Environmental predictor variables were the distance of the capture place from the forest edge (m), forest category (continuous forest, forest fragment), forest size (ha), percent edge habitat of a fragment (surface in close proximity [≤50 m] to the forest edge in relation to total fragment size [cf. Kiene et al., 2020]), vegetation clearance (PC1), and forest maturation (PC2; vegetation and human disturbance data represented by principal components). A previous study has shown that the four host species do not react equally to forest fragmentation (Andriatsitohaina et al., 2020). While three hosts were mostly captured in fragments, *M. ravelobensis* was predominantly captured in continuous forest sites. Since such host‐specific habitat preferences may impact or mask the effects of other factors on the parasites, host species*forest category (continuous forest vs. forest fragment) was included as an interaction term in all relevant models. The data are publicly accessible in the Zenodo repository (https://doi.org/10.5281/zenodo.4297519). Since sampling location (ANK, MAR), sampling year (2017, 2018), and month (May, June, July, August, September, October) were found to be associated with differences in parasite prevalence (data not shown), they were integrated as random factors into all models to control for possible and confounding spatiotemporal dynamics in the dataset. Using them as predictor variables was beyond the scope of this study and also not possible due to the heterogeneous sampling strategy across region, year, and month (i.e., the two regions were not sampled across all months in both years) which precluded their systematic analysis. However, we made sure that at any time we studied continuous forest sites and fragment sites in parallel to preclude systematic seasonal biases in the parasite dataset.

Host density data were square‐root‐transformed, and forest size and distance to edge data were log‐transformed to achieve normal distribution. Presence–absence data of parasite morphotypes are binomial by definition and the logit‐link was therefore used in the respective models. Models concerning the GPSR were based on Poisson assumption and used log‐link. Since some of the predictor variables could only be calculated for hosts from forest fragments, while others were available for the complete dataset, two sets of global models were built, one for the complete dataset and one for the fragment dataset (Table 2). Some numerical predictor variables were correlated with each other (Table 3) and were therefore never tested together in one global model. Therefore, three different global models were built for each dataset. As a consequence, six global models were built for each dependent variable (models A‐C: complete dataset, models D‐F: fragment dataset, Table 2).

**TABLE 2:** Composition of six different global models, fitted for each endoparasite taxon and the gastrointestinal parasite species richness (GPSR) as dependent variables separately

	Dataset	Fixed factors	Random factors
model A	all host individuals	host sex + host species + body condition + cont. versus frag. + host species*cont. versus frag. + vegetation clearance	sampling site + sampling year + month
model B		host sex + host species + body condition + cont. versus frag. + host species*cont. versus frag. + forest size + forest maturation	
model C		host sex + host species + cont. versus frag. + host species*cont. versus frag. + host density + distance to edge	
model D	all hosts from forest fragments	host sex + host species + host density + edge percentage	sampling site + sampling year + month
model E		host sex + host species + body condition + forest size + vegetation clearance	
model F		host sex + host species + distance to edge + forest maturation	

Abbreviations: Cont., continuous forest; frag., forest fragment.

**TABLE 3:** Pearson correlation coefficients and their significance for all continuous factor combinations analyzed with (a) the dataset on all host individuals, and (b) the dataset on hosts from forest fragments

Dataset for Models A‐C (all host individuals)						
*n* = 835 p* < .0033	Host density	Body condition	Forest size	Distance to edge	Vegetation clearance	Forest maturation
Host density		−0.02	0.19	0.29	0.12	−0.42
Body condition	0.5395		0.03	−0.10	0.10	0.00
Forest size	**<0.0001**	0.3511		0.69	−0.43	−0.22
Distance to edge	0.0958	0.0067	**<0.0001**		−0.49	−0.26
Vegetation clearance	**0.0009**	0.3429	**<0.0001**	**<0.0001**		0.09
Forest maturation	**<0.0001**	0.9928	0.0631	**<0.0001**	0.0120	

Dataset for Models D‐F (all hosts from forest fragments)							
*n* = 465 p* < .0024	Host density	Body condition	Forest size	Distance to edge	Edge percentage	Vegetation clearance	Forest maturation
Host density		0.03	0.13	−0.06	0.14	−0.31	−0.48
Body condition	0.5096		0.10	0.15	−0.16	0.02	0.01
Forest size	0.0090	0.0321		0.43	−0.64	−0.04	−0.08
Distance to edge	0.1808	**0.0014**	**<0.0001**		−0.54	0.27	0.09
Edge percentage	0.2903	**0.0007**	**<0.0001**	**<0.0001**		−0.47	−0.32
Vegetation clearance	**<0.0001**	0.6441	0.4163	**<0.0001**	**<0.0001**		0.48
Forest maturation	**<0.0001**	0.9027	0.0898	0.0513	**<0.0001**	**<0.0001**	

Note

Pearson correlation coefficient: above the diagonal, associated *p*‐values: below the diagonal. Significant correlations are highlighted in bold. p* = adjusted level of significance after Bonferroni correction.

The selection of the best model from a set of multiple candidate models (derived from each global model) was based on the Akaike information criterion (AIC) as described by Burnham and Anderson (2002) using the corrected AIC (AICc) to compensate for small sample sizes (Hurvich & Tsai, 1989). The best model with the highest statistical support and the ones with similarly low AICc values (∆AICc < 2) were considered for interpretation of content. To obtain them, all possible combinations of variables included in each global model were computed with the model selection function “dredge()” of the R‐package “MuMIn” (Barton, 2018. MuMIn: Multi‐model inference. R‐package version 0.12.2/r18. http://R‐Forge.R‐project.org/projects/mumin/) and ranked according to their AICc values. Host species were compared in post hoc tests (Tukey tests, R‐package “multcomp”; Hothorn et al., 2008) whenever the species parameter was significant.

## 3 RESULTS

**FIGURE 3:**
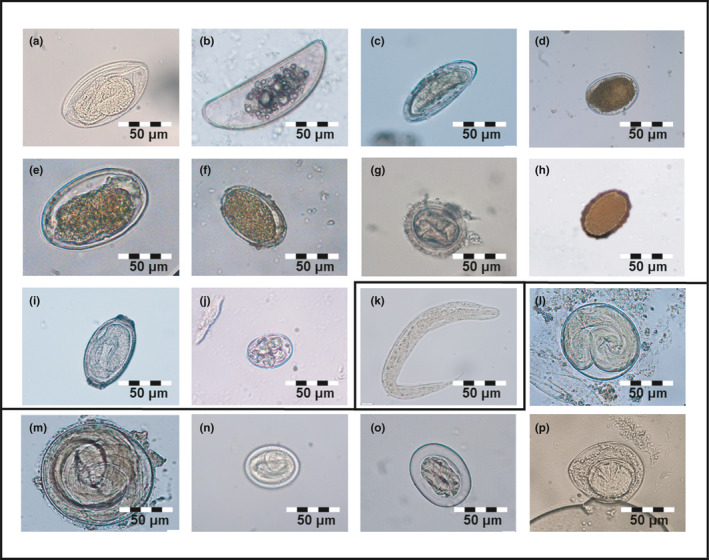
Gastrointestinal parasite morphotypes discriminated in this study. Excreting host species are abbreviated as follows: Mm = *M. murinus*; Mr = *M. ravelobensis*; Em = *E. myoxinus*; Rr = *R. rattus*. (a) Enterobiinae gen. sp.—Mm, Mr, Rr; (b) *Lemuricola* sp.—Mm, Mr, Rr; (c) *Syphacia* sp.—Rr; (d) strongyle egg 1—Mm, Mr; (e) strongyle egg 2—Mr, Rr; (f) strongyle egg 3—Rr; (g) ascarid egg 1—Mm, Mr; (h) ascarid egg 2—Mr; (i) *Trichosomoides crassicauda*—Rr; (j) Eimeriidae gen. sp.—Mr.; (k) *Strongyloides* sp.—Mm, Mr, Rr; (l) Subuluroidea fam. gen. spp.—all host species; (m) Subuluroidea‐like egg—Em, Rr; (n) spirurid egg 1—all host species; (o) spirurid egg 2—Rr; (p) *Hymenolepis* sp.*—*Mm, Mr; a‐j: homoxenous life cycle, k: homoxenous life cycle with homogenic or heterogenic free‐living development, l‐p: heteroxenous life cycle

**FIGURE 4:**
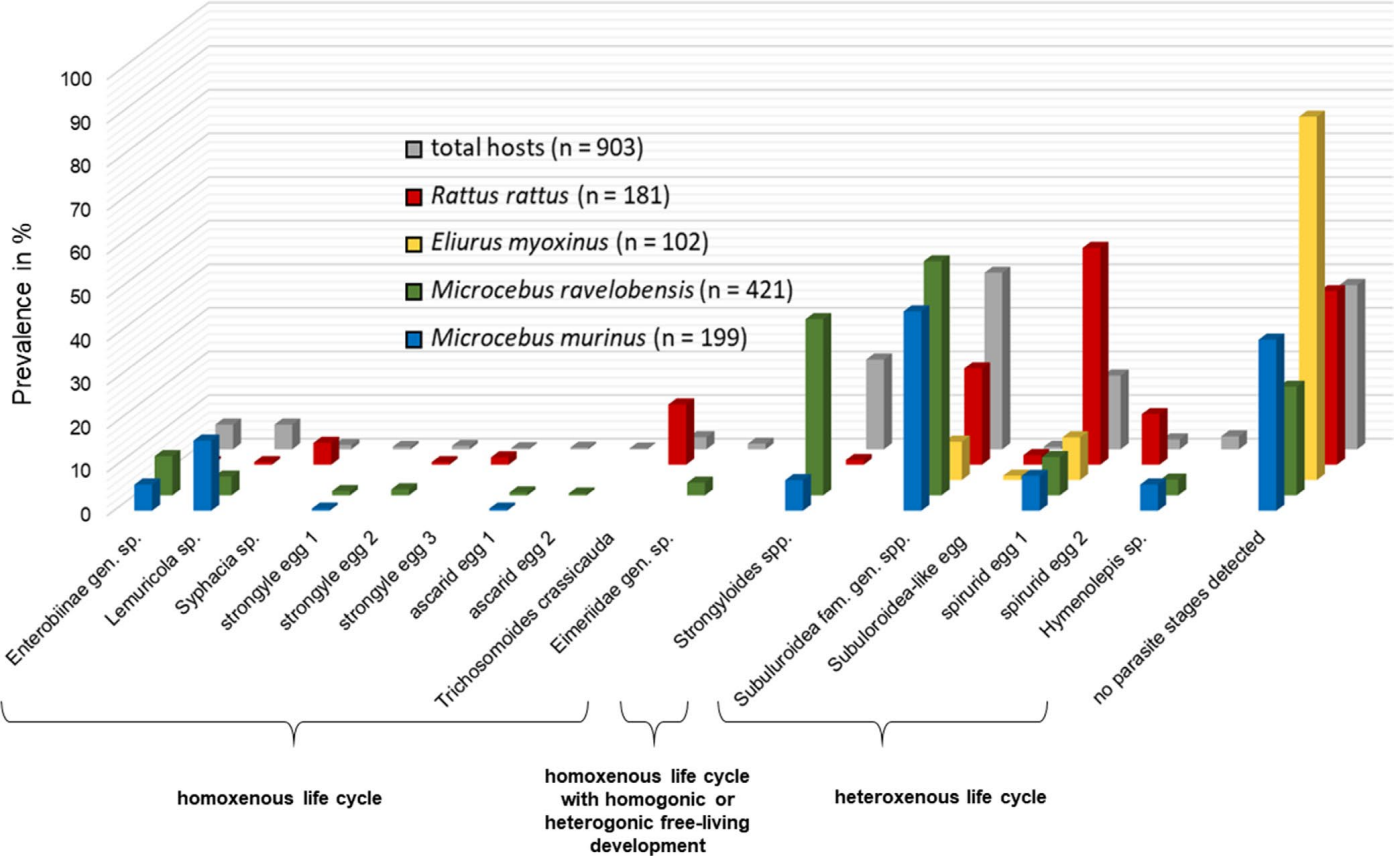
Total and host‐specific prevalences (in %) of the 16 gastrointestinal parasite morphotypes and the proportion of noninfected individuals in the four host species

A total of 1,418 fecal samples from 903 individual hosts were screened for parasite stages. Hosts comprised 199 *M. murinus*, 421 *M. ravelobensis*, 102 *E. myoxinus*, and 181 *R. rattus* trapped in continuous and fragmented forests (Table 4). Across all host species, 16 different parasite morphotypes (13 nematode eggs, one nematode larva, one cestode egg, one protozoan oocyst) could be discriminated (Figure 3). The majority of primate hosts (70.5%, 437/620) but less than half (44.5%, 126/283) of the rodent hosts were positive for at least one gastrointestinal parasite morphotype. Overall prevalences for primates were 58.3% (141/242) in forest fragments and 78.3% (296/378) in continuous forest sites, while rodent overall prevalences were 48.4% (108/223) in forest fragments but only 30.0% (18/60) in continuous forest sites. Between 3 and 11 parasite morphotypes were found in each host species (Figure 4; File S3). Individual hosts excreted up to six different parasite morphotypes simultaneously with an overall mean GPSR of 1.027 (σ = 1.013) and varied between 0.196 (σ = 0.443) in *E. myoxinus* and 1.261 (σ = 1.123) in *M. murinus* (File S3). Host‐related and environment‐related inferences by GLMMs were feasible only for the five most prevalent morphotypes (Enterobiinae gen sp., *Lemuricola* sp., *Strongyloides* sp., Subuluroidea fam. gen. spp., spirurid egg 1; Figure 4). A guiding overview on the results of the best models with lowest AICc is provided in Table 5, while modeling results are summarized in the text for all submodels with ∆AICc < 2, and details of all submodels with ∆AICc < 2 are documented in File S4.

**TABLE 4:** Number of hosts captured in the continuous and fragmented forest sites

	*M. murinus*	*M. ravelobensis*	*R. rattus*	*E. myoxinus*	Total
Continuous forest	42 (21%)	336 (80%)	34 (19%)	26 (25%)	438 (49%)
Fragmented forest	157 (79%)	85 (20%)	147 (81%)	76 (75%)	465 (51%)
Total	199 (100%)	421 (100%)	181 (100%)	102 (100%)	903 (100%)

**TABLE 5:** Summary of the results (significance and directionality) of the best models that were derived from each global model by means of the corrected Akaike's Information Criterion (AIC_c_) (Burnham & Anderson, 2002)

Parasite morphotype/GPSR	Host data		Model		Host‐related factors						Habitat‐related factors								
					Sex				Species	Body condition	Density	Host/forest category interaction	Forest category (cont. vs. frag.)		Forest size (log ha)	Distance from edge	Percent edge area	Vegetation clearance	Forest maturation
Parasites with a homoxenous life cycle (one host per life cycle)																			
Enterobiinae gen. sp.	all		A		NS				**Mr >Rr**										
	all		B		NS				**Mr >Rr**										**neg**.
	all		C						**Mr >Rr**										
	frag.		D						**Mr >Rr**								NS		
	frag.		E		NS				**Mr >Rr**										
	frag.		F		NS				**Mr >Rr**										
*Lemuricola* sp.	all		A		NS				**Mm >Mr, Rr, Em**	NS									
	all		B		NS				**Mm >Mr, Rr, Em**	NS									**pos**.
	all		C		**♂ > ♀**				**Mm >Mr > Rr, Em**										
	frag.		D						NS										
	frag.		E						NS					NS					
	frag.		F		NS				NS										**pos**.
Homoxenous parasites with homogenic or heterogenic free‐living development (one host per life cycle, but parasites can undergo free‐living generations between parasitic generations)																			
*Strongyloides* spp.		all		A			**Mr >Mm, Rr, Em**						**cont.>frag**.					**neg**.	
		all		B			**Mr >Mm, Rr, Em**						**cont.>frag**.	NS					
		all		C			**Mr >Mm > Rr, Em**						NS			**pos**.			
		frag.		D			**Mr >Mm, Rr, Em**				NS								
		frag.		E			**Mr >Rr, Em**							**pos**.					
		frag.		F			**Mr >Mm, Rr, Em**									NS			
Parasites with a heteroxenous life cycle (intermediate host required)																			
Subuluroidea fam. gen. spp.		all		A			**Mm, Mr >Rr**					NS	NS					**neg**.	
		all		B			**Mm, Mr >Rr**					NS		NS					
		all		C			**Mm, Mr >Rr**					NS	NS			**pos**.			
		frag.		D			**Mm, Mr, Rr >Em; Mm >Rr**										**neg**.		
		frag.		E			**Mm, Mr >Em; Mm >Rr**							NS				**neg**.	
		frag.		F			**Mm, Mr >Em; Mm >Rr**									**pos**.			
spirurid egg 1		all		A			**Rr >Mr**			**pos**.		NS	NS					**neg**.	
		all		B			**Rr >Em, Mm, Mr**			**pos**.									
		all		C			**Rr >Em, Mm, Mr**						NS			NS			
		frag.		D			**Rr >Em, Mm, Mr**										NS		
		frag.		E			**Rr >Em, Mm, Mr**			**pos**.								**neg**.	
		frag.		F			**Rr >Em, Mm, Mr**												
Gastrointestinal parasite species richness																			
GPSR		all		A				**Mm, Mr, Rr >Em; Mr >Rr**		**pos**.		NS	NS					**neg**.	
		all		B				**Mm, Mr, Rr >Em; Mr >Rr**		**pos**.		NS	NS	NS					
		all		C				**Mm, Mr, Rr >Em; Mr >Rr**				NS	NS			**pos**.			
		frag.		D				**Mm, Mr, Rr >Em**									**neg**.		
		frag.		E				**Mm, Mr, Rr >Em**		**pos**.								**neg**.	
		frag.		F				**Mm, Mr, Rr >Em**								**pos**.			

Note

Factors not part of a global model are left blank, factors removed from the global model in the process of model selection are framed and marked in light gray, and factors being part of the best model are framed and marked in dark gray. Significant effects are highlighted in bold. GPSR =gastrointestinal parasite species richness; cont. = continuous; frag. = fragmented; **♂** = male hosts; **♀** = female hosts; pos. = positive effect; neg. = negative effect; NS = not significant; Mm = *M. murinus*; Mr = *M. ravelobensis*; Em = *E. myoxinus*; Rr = *R. rattus*.

### 3.1 Parasites with a homoxenous life cycle

#### 3.1.1 Oxyuridae

Three parasite morphotypes were determined as members of the Oxyuridae family. The first morphotype, Enterobiinae gen. sp. (Figure 3a) amounted to a total prevalence of 5.7% (51/903) and was primarily excreted by the two *Microcebus* spp. (6.0% and 9.0%), and by one *R. rattus* individual (Figure 4). GLMMs revealed significantly higher infection rates in *M. ravelobensis* compared with *R. rattus* (all models; estimates: 2.51–2.90, *p*‐values ≤.02). Forest maturation was negatively associated with this egg morphotype in the complete dataset (model B; estimates: −0.39 to −0.36, *p*‐values ≤.03), but not in the fragment dataset.

The second oxyurid morphotype, morphologically determined as *Lemuricola* sp. (Figure 3b), showed a total prevalence of 5.7% (51/903) and was again primarily excreted by the two *Microcebus* spp. (16.1% and 4.3%), but also by one *R. rattus* individual (Figure 4; File S3). GLMMs with the complete dataset showed that *M. ravelobensis* and *R. rattus* were significantly less often infected than *M. murinus* (models A‐C; estimates: −3.71 to −0.98, *p*‐values ≤.04). According to our expectation, three best submodels (obtained from global models B and C) revealed significantly higher prevalences in male than in female hosts (estimates: 0.65–0.71, *p*‐values ≤.05). Additionally, one of six best models of global model B showed a positive relationship between good body condition and *Lemuricola* spp. prevalence (estimate = 0.93, *p*‐value = .04). Regarding habitat‐related factors, forest maturation was positively associated with the presence of *Lemuricola* spp. (models B and F; estimates: 0.45–0.79, *p*‐values ≤.04).

The third morphotype, which was exclusively found in *R. rattus* (5.0%; total prevalence: 1% [9/903]), was identified as *Syphacia* sp. (Figure 3c). Due to low prevalence, ecological modeling could not be conducted.

#### 3.1.2 Strongylida

**FIGURE 5:**
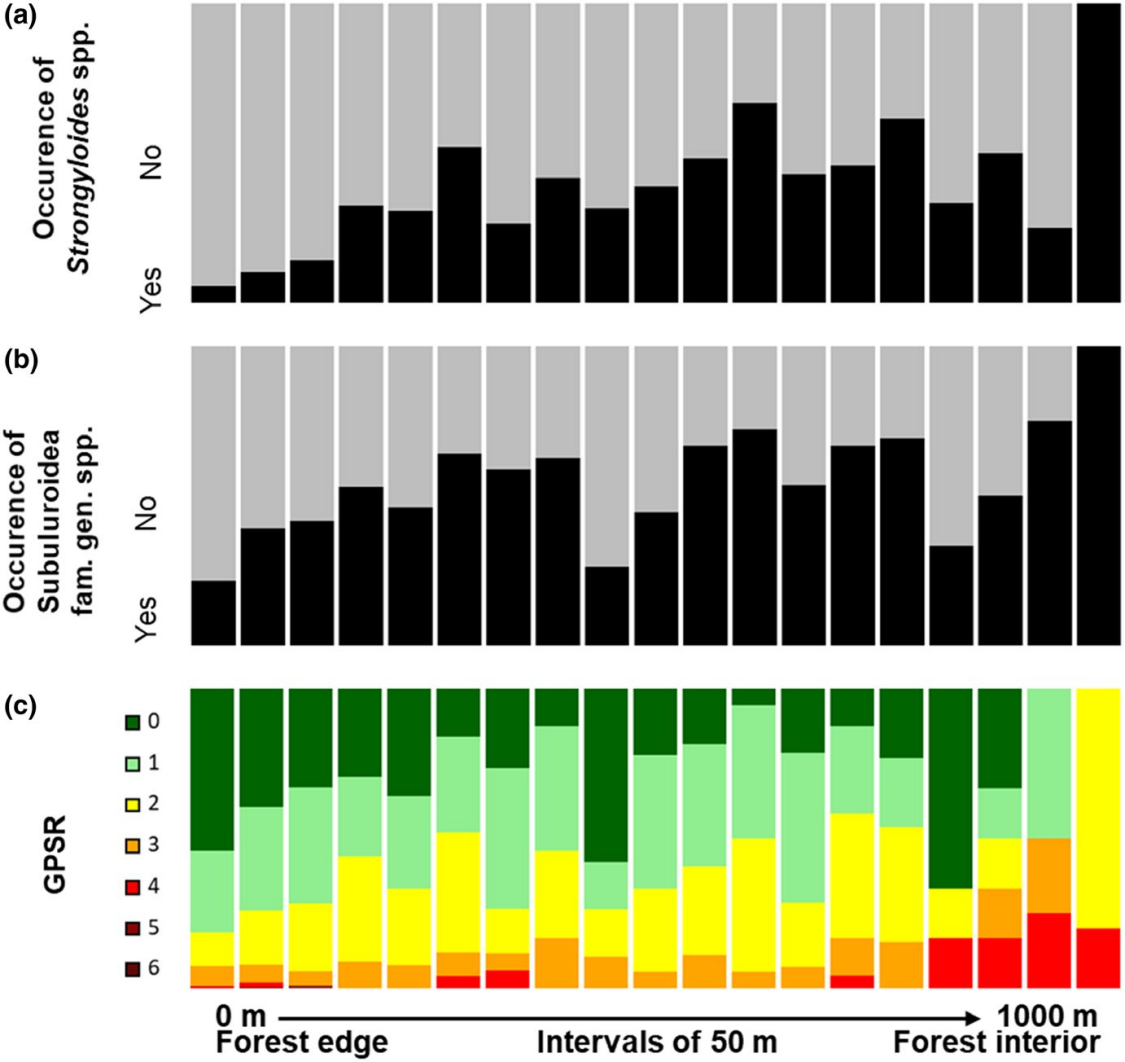
Proportion of infected versus noninfected fecal samples for significantly impacted parasite morphotypes (parts a–b) and distribution of gastrointestinal parasite species richness (GPSR; part c) across a distance gradient from the forest edge (0 m, left end) to the forest interior (1,000 m, right end). Each column corresponds to one 50 m forest segment along the distance gradient. The relative proportion of fecal samples with simultaneous presence of 0–6 different parasite morphotypes (GPSR) is marked by different colors within each column (part c)

Three parasite morphotypes (strongyle eggs 1–3, Figure 3d–f) were morphologically assigned to this order. These morphotypes revealed generally low total prevalences of 0.3%–0.8% (3–7/903) and were excreted by either the two mouse lemurs (strongyle egg 1), by *M. ravelobensis* and *R. rattus* (strongyle egg 2), or by *R. rattus* alone (strongyle egg 3; Figure 5, details in File S3). Due to the sporadic appearance of these eggs, ecological modeling could not be performed.

#### 3.1.3 Ascarididae

Two parasite morphotypes were morphologically assigned to this family. Ascarid eggs 1 and 2 (Figure 3g,h) were detected only in the two mouse lemurs or *M. ravelobensis* alone (Figure 4). Again, host‐specific and total prevalences were low with 0.4% (4/903) and 0.2% (2/903), respectively. It is very likely that the two ascarid egg morphotypes belong to the same species; however, genetic allocation was impaired by insufficient egg quantity. Similarly, prevalences were too low for reliable ecological modeling.

#### 3.1.4 Trichosomoides

One egg morphotype, excreted only by *R. rattus* (13.8%; total prevalence: 2.8% [25/903]), was morphologically identified as *Trichosomoides crassicauda* (Figure 3i). The eggs of this bladder parasite are excreted with the urine and thus likely contaminated the feces in the traps. The low overall prevalence precluded ecological modeling.

#### 3.1.5 Protozoa

One protozoan morphotype, namely coccidian oocysts, was morphologically determined as Eimeriidae gen. sp. (Figure 3j) and was only detected in *M. ravelobensis* (2.9%; total prevalence: 1.33% [12/903]). Ecological modeling was not possible for this taxon due to low prevalence.

### 3.2 Homoxenous parasites with homogenic or heterogenic free‐living development

#### 3.2.1 Strongyloides

*Strongyloides* spp. larvae (Figure 3k) were morphologically determined and revealed a total prevalence of 20.6% (186/903). They were mainly found in the feces of *M. ravelobensis* (40.4%), but also in *M. murinus* and *R. rattus* (7.0% and 1.1%; Figure 4; File S3). GLMMs showed a significantly higher prevalence in *M. ravelobensis* than in the other hosts (all models; estimates: 1.41–4.20, *p*‐values ≤.01). As expected, prevalences were significantly lower in forest fragments than in the continuous forests (model A and B; estimates: −2.36 to −0.92, *p*‐values ≤.01). In addition, prevalence increased with increasing forest size in one of the best submodels (global model E, estimate = 0.45, *p*‐value = .04). Congruently, prevalences also increased with increasing distance to the forest edge (model C; estimates: 0.40–0.52, *p*‐values < .01; Figure 5a), but decreased with increasing vegetation clearance (model A; estimates: −0.24 to −0.23, *p*‐values ≤.04) in the complete dataset.

### 3.3 Parasites with a heteroxenous life cycle

#### 3.3.1 Subuluroidea

Two parasite morphotypes were morphologically assigned to this superfamily. The first morphotype, Subuluroidea fam. gen. spp. (Figure 3l) was shed by all host species. The total prevalence amounted to 40.5% (366/903), with highest infection rates in the two *Microcebus* species (53.7% and 45.7%) and lowest prevalence in *E. myoxinus* (8.8%; Figure 4, File S3). Genetic analyses revealed that eggs of this morphotype obtained from the two mouse lemur hosts most likely belong to the same species, while the two rodents harbored different species, which probably belong even to different genera.

GLMMs showed that prevalences were significantly lower in rodents than in mouse lemurs (all models; estimates_rodents‐mouse lemurs_: −1.94 to −1.41, *p*‐values ≤.04, estimates_mouse lemurs‐rodents_: 1.11–2.03, *p*‐values ≤.01). According to our expectations, prevalences significantly increased with increasing distance to the edge (models C and F; estimates: 0.50–0.74, *p*‐values <.001; Figure 5b) and decreased significantly within fragments with a higher percentage of edge habitat (model D; estimates = −2.66, *p*‐values <.001) as well as with increasing vegetation clearance (models A and E; estimates: −0.42 to −0.19, *p*‐values ≤.05).

The second egg type attributed to this superfamily, a Subuluroidea‐like egg (Figure 3m) with a total prevalence of 0.6% (5/903), was excreted by *R. rattus* and *E. myoxinus* only (Figure 4; File S3). Prevalences did not allow reliable ecological modeling.

#### 3.3.2 Spiruromorpha

Two parasite egg morphotypes were assigned to this infraorder. The first morphotype, spirurid egg 1 (Figure 3n), showed a total prevalence of 16.9% (153/903) and was excreted by all host species. Of these, *R. rattus* exhibited the highest prevalence with 49.7%, while the other host species ranged between 8.0% and 9.8% (Figure 4; File S3). Genetic investigations on eggs and adult worms collected from *R. rattus* feces suggest the presence of up to four Spiruromorpha species in this host species alone. Among these, *Protospirura muricola* is assumed and *Gongylonema neoplasticum* could be confirmed (Costa Cordeiro et al., 2018).

GLMMs showed that *R. rattus* was significantly more often infected than the other host species (all models; estimates_other hosts‐_ *_R. rattus_*: −1.86 to −1.23, *p*‐values ≤.04, estimates_R. rattus‐other hosts_: 2.19–2.85, *p*‐values ≤.001), and hosts with generally better body condition exhibited significantly higher prevalences (model A, B, and E; estimates: 0.62–1.36, *p*‐values ≤.03). In contrast to our predictions, infection rates were higher in fragmented than continuous forests in one submodel (global model A, estimate = 0.67, *p*‐value = .04). Finally, and in line with our expectations, there was a negative impact of increasing vegetation clearance on infection rates (models A and E; estimates: −0.55 to −0.41, *p*‐values <.01).

The second morphotype, spirurid egg 2 (Figure 3o), was morphologically determined and was only shed by *R. rattus* (11.6%; total prevalence: 2.3% [21/903]). Ecological modeling could not be conducted due to low prevalences.

#### 3.3.3 Cestodes

One cestode egg morphotype was found and morphologically identified as *Hymenolepis* sp. (Figure 3p). Egg excretion occurred in the two mouse lemur species (3.6%–6.0%; Figure 4; File S3) and amounted to a total prevalence of 3.0% (27/903). Ecological modeling was not possible due to low prevalences.

### 3.4 Gastrointestinal parasite species richness (GPSR)

Mouse lemurs and black rats had significantly higher GPSRs than *E. myoxinus* (all models; estimates: 0.70–1.76, *p*‐values ≤.03). Moreover, *M. ravelobensis* had a higher GPSR than *R. rattus* in the complete dataset (models A‐C; estimates: 0.55–0.62, *p*‐values ≤.01). Furthermore, body condition was positively associated with GPSR (models A, B, and E; estimates: 0.20–0.41, *p*‐values ≤.04). Regarding habitat‐related factors, increasing distance to the forest edge had a positive effect on GPSR (models C and F; estimates 0.15–0.19, *p*‐values <.01; Figure 5c). Consistently and in line with our hypotheses, a higher percentage of edge habitat (model D; estimates: −0.88 to −0.87, *p*‐values <.01) and vegetation clearance had a negative effect on GPSR (models A and E; estimates: −0.13 to −0.12, *p*‐values ≤.02). Although the factor forest category had no significant impact in general, the interaction term host species*forest category revealed a significantly higher GPSR in continuous than fragmented forests in one submodel, but only for *M. ravelobensis* (global model B, estimate = 0.63, *p*‐value = .03).

## 4 DISCUSSION

Gastrointestinal parasites constitute an important part of the world's biodiversity which is illustrated by this study identifying 16 parasite morphotypes and at least 21 parasite taxa parasitizing the four studied host species. Like their hosts, gastrointestinal parasites may be vulnerable to habitat fragmentation and degradation. However, this relationship remained so far largely unexplored. In this study, we compared and integrated the effects of forest fragmentation on gastrointestinal parasites in four small mammal species from northwestern Madagascar. Since the basic context for understanding the links between parasite and environmental changes is provided by the respective host–parasite interactions, impacts of host‐related factors such as host lifestyle, population density, body condition, and sex were also assessed and will be discussed first. Indeed, different gastrointestinal parasite taxa were affected by environmental and host‐related factors in different ways. However, integrative modeling was only possible for the five most abundant parasite morphotypes, limiting a more detailed discussion of possible cause–effect relationships to them.

### 4.1 Gastrointestinal parasite infections in different host species

**FIGURE 6:**
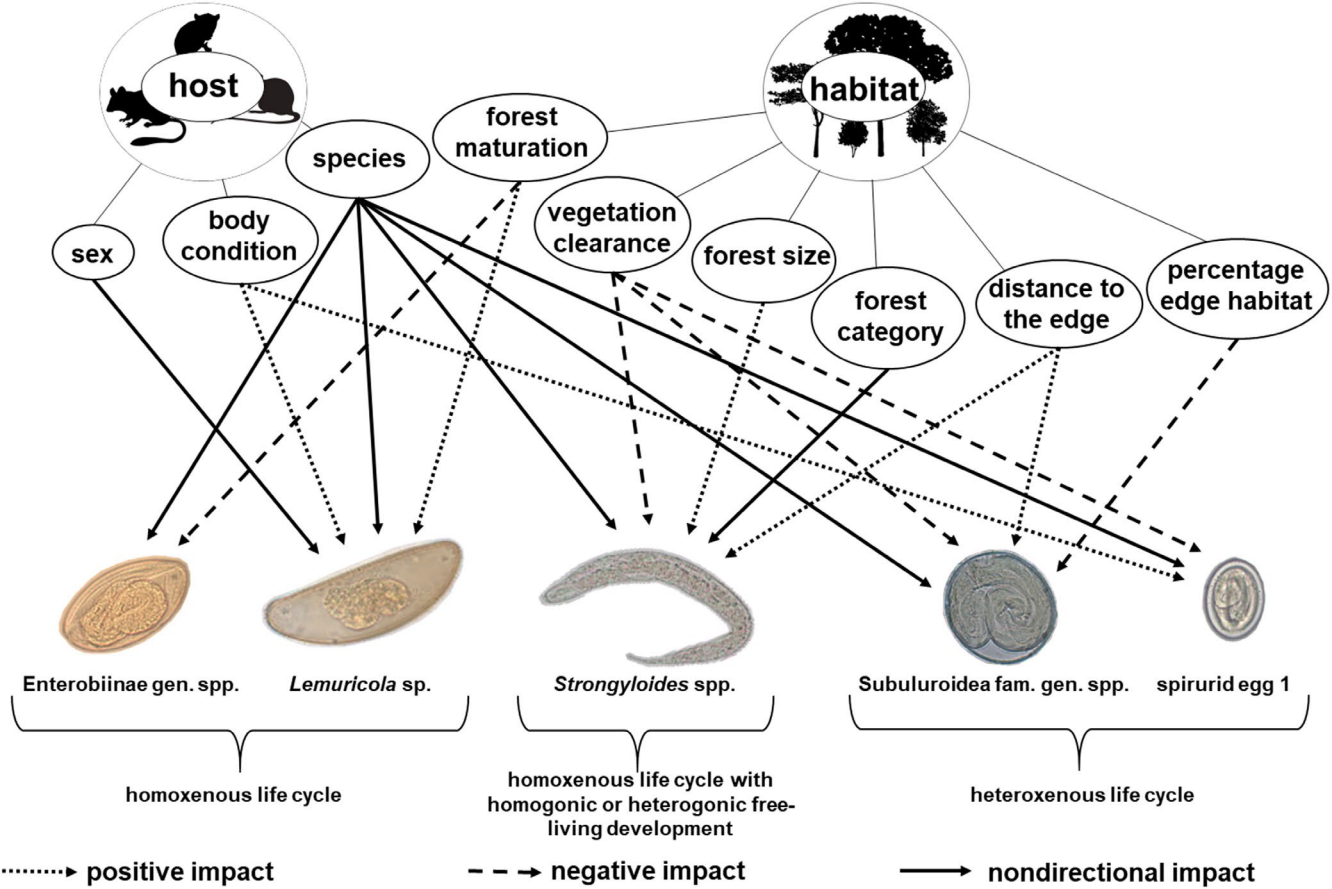
Schematic overview of factors impacting gastrointestinal parasite morphotype prevalences. Host‐related factors: species (*M. murinus*, *M. ravelobensis*, *E. myoxinus*, *R. rattus*), host sex, host body condition; habitat‐related factors: distance of host capture site from the forest edge (m), forest category (continuous vs. fragmented forest), forest size (ha), percent edge habitat (surface in close proximity [≤50 m] to the forest edge in relation to total fragment size), vegetation clearance (PC1), and forest maturation (PC2)

Significant prevalence differences between host species were shown for all five modeled parasite morphotypes as well as GPSR (Figures 4 and 6). The host's infection risk and consequently parasite prevalence is generally mediated by two‐factor categories. Firstly, by the abundance of infective parasite stages in the environment or potential intermediate hosts, their transmission routes, and host specificity, and secondly, by host accessibility to the parasite, which is often related to host lifestyle (e.g., social behavior, diet preferences) and host abundance (see section below).

Oxyurid nematodes, of which Enterobiinae gen. sp. and *Lemuricola* sp. could be modeled, have a homoxenous life cycle and therefore need only on a single host for their development. A common feature of most oxyurids is egg deposition around the anus, facilitating autoinfection and intraspecies transmission during close contact, but impeding interspecies transmission (Baker, 2008; Hugot et al., 1999). Here, both mentioned morphotypes were detected in the two mouse lemur species. Additionally, the morphotypes were each found in one *R. rattus* individual. Since at least for the genus *Lemuricola*, host specificity for hosts of the family Cheirogaleidae is described (Hugot et al., 1999; Irwin & Raharison, 2009); these may, however, have resulted from gastrointestinal passage instead of patent infections or a confusion with *Syphacia* eggs.

*Lemuricola* prevalences differed significantly between the two mouse lemur species. The higher prevalences in *M. murinus* than in *M. ravelobensis* might result from different sleeping site preferences. Wooden tree holes, as preferred by *M. murinus* (Ehresmann, 2000; Radespiel et al., 2003), are protected from weather influences and used over longer periods, promoting reinfections and parasite exchange between cosleepers up to several weeks or even months. In contrast, *M*. *ravelobensis* often uses more open sites or self‐built leaf nests (Radespiel et al., 2003; Thorén et al., 2010), which are more exposed to weather conditions, used less frequently and for shorter periods (Radespiel et al., 2003), possibly making spreading of *Lemuricola* sp. among *M. ravelobensis* less effective.

*Strongyloides* spp. also have a homoxenous life cycle. In this case, however, the prevalence in *M. ravelobensis* was high (>40%), while *M. murinus* and *R. rattus* showed much lower prevalences of 7.0% and 1.1%, respectively. Again, behavioral differences between host species might have caused these differences in infection risk. Besides potential perianal autoinfection (known for *Strongyloides stercoralis*, Olsen et al., 2009) and lactogenic infection of the offspring (Viney & Lok, 2015), a striking feature of this genus is that larvae in the environment can either develop into infective third‐stage larvae (homogenic development) or initiate a free‐living generation (heterogenic development), whose progeny develop into infective larvae (Zhou et al., 2019). Hosts are typically infected percutaneously and, more rarely, orally during contact with the soil. A preliminary study did not show significant differences between *M. murinus* and *M. ravelobensis* in foraging on the ground (Radespiel et al., 2006). However, *M. ravelobensis* are known for an opportunistic choice of sleeping sites which can even have contact to the soil, for example, under a pile of leaves or in small holes in the ground (Radespiel et al., 2003). Such sleeping habits, even if soil‐contact sites are visited only occasionally, could lead to higher infection rates in this species, but this hypothesis requires further validation.

All investigated host species excreted the egg morphotypes Subuluroidea fam. gen. spp. and spirurid egg 1, representing nematodes with a heteroxenous life cycle depending on arthropods as intermediate hosts which are typically ingested as part of the diet. However, and in contrast to our expectations, the frugivorous *E. myoxinus* also harbored this egg morphotype. Most likely, *E. myoxinus* ingests intermediate arthropod hosts at relatively low rates and possibly accidentally (prevalences between 8.8% and 9.8%) while feeding on fruits, and the respective parasite species appear to have adapted to this native Malagasy rodent. Similarly, the spirurid *P. muricola* was found to have adapted to the vegetarian rodent *Otomys tropicalis*, the tropical vlei rat (Smales et al., 2009).

However, the prevalence of the Subuluroidea fam. gen. spp. egg morphotype also differed between the three omnivorous host species and was more than doubled in the two mouse lemurs (45.7% and 53.7%) compared with *R. rattus* (22.1%), while the spirurid egg 1 morphotype occurred much more frequently in *R. rattus* (49.7%) than in the two mouse lemur species (about 8% each). Such differences in Subuluroidea fam. gen. spp. prevalence between mouse lemurs and *R. rattus* may be attributed to the high phylogenetic distance between lemurs and rodents. In fact, it could be shown that Subuluroidea fam. gen. spp. that infected mouse lemurs and rodents belonged to different species. In addition, differences may also be explained by different food preferences.

In contrast, it remains unclear whether mouse lemurs and rodents carried different or the same spirurid species (all carried the spirurid egg 1 morphotype), since a broad variety of Spiruromorpha species produces eggs with similar morphology (Baker, 2008). The simultaneous presence of several spirurid species was, however, confirmed for *R. rattus*. One likely species was *Protospirura muricola*, known to occur primarily in rodents in Africa, Southeast Asia, and Central and South America (Smales et al., 2009), but capable of infecting also primates (Kouassi et al., 2015; Petrzelkova et al., 2006; Smales et al., 2009). *P. muricola* may hence also have parasitized the mouse lemurs and *E. myoxinus* in our study. Smales et al. (2009) reported that occurrences of *P. muricola* outside Africa could always be traced back to the cosmopolitan rodents *R. rattus* and *R. norvegicus*. The significant prevalence differences between the invasive *R. rattus* and the native host species may then be attributed to *R. rattus*, which might have introduced and may continue to spread the parasite into the ecosystem serving as a reservoir. The second species detected in *R. rattus*, *Gongylonema neoplasticum,* was in contrast reported to be restricted to rats of the genus *Rattus* (Setsuda et al., 2018). This species may therefore have contributed to the spirurid eggs 1 in *R. rattus*, but presumably not in the other host species.

Differences in parasite species richness between the four host species may at least partially result from differences in host socioecology. *E. myoxinus* exhibited the lowest, while *M. ravelobensis* showed the highest mean GPSR (File S3) which is concordant to the different number of parasite morphotypes found within each species (*E. myoxinus*: 3, *M. murinus*: 8, *R. rattus*: 11, *M. ravelobensis*: 11). These species differences may be mainly explained again by differences in the diet (frugivorous vs. omnivorous, see above). Springer and Kappeler (2016) found indeed a similar pattern of higher numbers of gastrointestinal parasites with heteroxenous life cycles in omnivorous (*Microcebus berthae*, *M. murinus*, *Cheirogaleus medius*, *Mirza coquereli*) compared with herbivorous Malagasy lemurs (*Eulemur rufifrons*, *Propithecus verreauxi*). In addition, *E. myoxinus* is not only a solitary forager, but also sleeps solitarily (Poor, 2005). The resulting lower contact rates certainly contributed to a reduced infection risk with directly transmitted parasites.

### 4.2 Effects of host population density, sex, and body condition on gastrointestinal parasites

Increasing host population density can lead to increasing parasite infection risk either indirectly by increased environmental contamination with infective stages or directly by increased interactions with conspecifics and transmission (e.g., oxyurid eggs deposited at the anus) during social contacts (Altizer et al., 2003; Chapman et al., 2006; Stringer & Linklater, 2015). However, our study did not reveal an impact of host population density on any detected gastrointestinal parasite morphotype. As the prevalence of parasites with heteroxenous life cycles (Subuluroidea fam. gen. spp. and spirurid egg 1) depends on a combination of definitive and intermediate host availability, data on the definitive host population density alone may not be sufficient to explain our results. However, parasites with homoxenous life cycles (Enterobiinae gen. sp., *Lemuricola* sp., and *Strongyloides* spp.) should be affected by host abundance (Arneberg et al., 1998). This lacking impact of host population density on the detected Oxyuridae may be explained by the social organization of the studied host species. Although mouse lemurs are known to forage alone, they spend the days sleeping in more or less stable matrilinear groups that do not vary much in size, since they split when becoming too large (Radespiel et al., 2001; Weidt et al., 2004). As matings are confined to a very limited time period of the year, most infections with oxyurids will take place between animals sleeping in close body contact or performing allogrooming with familiar individuals of the same social group (Eichmueller et al., 2013; Thorén et al., 2016). Under these conditions, infection risk may become mostly independent from population density, but may vary rather between different sleeping groups (Nunn et al., 2011). Regarding the soil‐transmitted *Strongyloides* spp., developing host immunity may account for the lacking effect of population density on increased parasite infection risk. For example, experimental infections of mice with as few as six *S. ratti* larvae produced marked resistance to reinfections by reducing larvae excretion during challenge infection by 97% (Dawkins & Grove, 1982).

In our modeling approach, we also considered two individual host traits generally assumed to impact the parasite infection risk, host sex, and body condition. An effect of host sex was only detected in *Lemuricola* sp., for which some models suggested higher infection rates for male hosts than for females. Sex differences in parasite prevalence of mouse lemurs have already been demonstrated in several studies, mostly on ectoparasites. In *Microcebus rufus*, males were shown to be exclusively responsible for sucking louse transmission (Zohdy et al., 2012) and also exhibited higher sucking louse and nematode prevalences (Rafalinirina et al., 2007). In our study population, male *M. murinus* and *M. ravelobensis* were also found to have higher sucking louse prevalences than females (Kiene et al., 2020). Higher parasite prevalences in males are typically explained by different sex hormone profiles, which can either stimulate (estrogens in females) or depress (androgens in males) immunity (Klein, 2004; Schalk & Forbes, 1997; Zuk & Mckean, 1996). However, the relationship between testosterone and immunity is certainly more complex, as for example positive effects of testosterone have also been reported (Ezenwa et al., 2012) and effects also seem to depend on the parasite type (Fuxjager et al., 2011). Sex differences in parasite infection risk are also often attributed to behavioral differences. Due to more extensive ranging patterns (Greenwood, 1980; Lawson Handley & Perrin, 2007; Radespiel, 2000) and more frequent “risk‐taking” behaviors, males are more likely to have social encounters, which may raise pathogen transmission risk (Kraus et al., 2008; Poirotte & Kappeler, 2019; Soliman et al., 2001; Zuk & Mckean, 1996). However, these mechanisms may probably act stronger on ectoparasite than on helminth infections (Schalk & Forbes, 1997).

In our study, the spirurid egg 1 morphotype prevalence as well as GPSR showed a positive relationship between body condition and parasite infections. In general, the host's body condition is subject to seasonal and ontogenetic plasticity. This implies that body condition may influence parasite infections, but parasite infections in turn may also influence body condition. The impact of parasitism on body condition is usually attributed to the competition for nutrients between host and parasite, but also to tissue damage resulting in organ malfunction and protein loss (Holmes, 1985). A dwindling food intake due to loss of appetite has also been associated with parasite infections (Arneberg et al., 1995; Fox, 1997; Ghai et al., 2015). Conversely, hosts in better body condition should rather be able to mobilize resources for the defense against pathogens than hosts in a worse situation (Bonneaud et al., 2003; Martin et al., 2003; Ujvari & Madsen, 2006). Both explanations would result in lower parasitism in animals with better body condition. The results of our study, however, correspond rather to the findings of Rafalinirina et al. (2007), who demonstrated that *M. rufus* in better body condition exhibited higher gastrointestinal parasite and ectoparasite prevalences. The ability of good quality hosts to sustain higher parasite loads was suggested by the author to explain these results. This “well‐fed host hypothesis” should, however, be especially applicable to ectoparasites (Christe et al., 2003; Hawlena et al., 2005). Due to an active infestation compared with the more passive infection mode by ingesting gastrointestinal parasites, ectoparasites could be attracted to hosts in good body condition (Christe et al., 2007). Instead, explanations for gastrointestinal parasites rather involve that such hosts might have a higher feeding capacity, increasing the probability of oral pathogen intake. In addition, older individuals may accumulate parasites over time if no protective immunity develops (Bellay et al., 2020).

### 4.3 Edge effects, fragmentation responses, and the impact of vegetation parameters on gastrointestinal parasites

Six environmental factors were used to infer effects of habitat fragmentation and structure on gastrointestinal parasites (Figure 6). While four factors (forest category, forest size, distance to the forest edge, percentage of edge habitat) are directly related to habitat fragmentation, the two others provide estimates of vegetation structure and human disturbance (i.e., vegetation clearance, forest maturation). The homoxenous oxyurids Enterobiinae gen. sp. and *Lemuricola* spp. were neither affected by habitat fragmentation parameters or edge effects nor vegetation clearance, which may be explained by their egg deposition at the host's anus (Taffs, 1976), providing a stable microenvironment for parasite development and survival despite altering macroenvironmental conditions. Nevertheless, the significant impact of forest maturation on prevalence of two oxyurids (Enterobiinae gen. sp., *Lemuricola* sp.) suggests that they are not entirely independent from environmental conditions. Intraspecies transmission occurs by oral uptake of infective eggs during interindividual contacts, mostly at highly frequented sheltered sleeping sites (Baker, 2008; Irwin & Raharison, 2009). Under these conditions, higher numbers of older trees in mature and pristine forests providing sheltered tree holes might have promoted *Lemuricola* sp. infection rates. This may have been particularly relevant for *M. murinus* which prefers wooden tree holes as sleeping sites and had the highest prevalence of all four host species.

In contrast and as expected, the other modeled parasite morphotypes, exhibiting quite different host external development and transmission modes compared with the oxyurids, were not affected by forest maturation, but vulnerable to habitat fragmentation and edge effects and/or vegetation clearance. The latter parameter can also be (among others) linked to forest edges. Several studies across different forest habitats worldwide revealed that vegetation differs depending on proximity to the edge (Chen et al., 1992; Laurance, 1991; Murcia, 1995; Nelson & Halpern, 2005). In the study region, the northwestern Malagasy dry forests, it has been demonstrated that tree stem density was lower in proximity to the edge compared with the forest interior (Malcolm et al., 2016). Such differences in vegetation structure may in turn lead to different levels of protection from weather influences like solar radiation or wind (Foggo et al., 2001). Resulting differences in environmental buffering effects can cause differences in microclimates at the edge compared with the forest interior, which may constrain or facilitate species survival or reproduction (Gehlhausen et al., 2000).

Here, the *Strongyloides* morphotype proved to be most vulnerable to environmental conditions as it was impacted by most habitat‐related factors indicative for forest fragmentation and degradation. This parasite genus showed significantly higher prevalences in continuous than in fragmented forests, and parasite infection risk increased with increasing forest size. Intriguingly, there was also a positive impact of the distance to forest edge and a negative effect of vegetation clearance on parasite infection risk which both suggest strong negative ecological edge effects on *Strongyloides* spp. These impacts are most likely related to the complex life cycle of *Strongyloides* spp., which are homoxenous parasites but can undergo heterogenic development with a free‐living generation in the environment (Baker, 2008; Eberhardt et al., 2007; Viney, 1999; Viney & Lok, 2015). Adverse influences of forest edges and vegetation clearance can be assumed for both homogenic and heterogenic cycles in terms of direct (e.g., ultraviolet damage or desiccation of developmental stages) and indirect (e.g., insufficient soil moisture) negative impacts on survival or reproduction, respectively.

Compared with *Strongyloides* spp., the heteroxenous Subuluroidea fam. gen. spp. and spirurid egg 1 morphotypes spend large parts of their life cycles outside the definitive hosts in an arthropod intermediate host (Baker, 2008; Irwin & Raharison, 2009). It could be assumed that these better protected morphotypes should be less affected by forest degradation‐related factors. However, vegetation clearance negatively impacted both morphotypes, and hosts captured in proximity to the forest edge and in forest fragments with proportionally larger edge habitat showed lower Subuluroidea fam. gen. spp. parasite infection risks. Interestingly, negative ecological edge effects in the study area have already been shown for some other arthropods, namely ectoparasites of the same host species (e.g., ticks or different mites, Kiene et al., 2020). The authors related these findings, among others, to abiotic factors, as ultraviolet radiation or humidity differ between the edge and interior of a forest (Kapos, 1989; Kiene et al., 2020; Murcia, 1995). Similar differences most likely apply to a degraded versus intact vegetation. Overall, it can be assumed that forest edges and vegetation clearance impacted the two heteroxenous parasite morphotypes not only in terms of negative effects on their eggs in the environment, but also by decreased arthropod intermediate host availability.

The GPSR was impacted by habitat‐related effects as well. Specifically, the significant impacts on GPSR mirror the edge‐related and vegetation clearance effects on *Strongyloides* spp. and the heteroxenous parasite morphotypes. Consequently, the gastrointestinal parasite diversity was lower in hosts living in open, degraded habitats or near forest edges, which can probably not maintain abiotic conditions favorable for the survival and development of soil‐transmitted gastrointestinal parasites and/or required intermediate arthropod hosts.

## 5 CONCLUSIONS

This study revealed a variety of impacts on gastrointestinal parasite infections that were partly host‐ and partly environment‐related. Thus, the initial expectation of gastrointestinal endoparasites being less susceptible to environmental changes than previously studied ectoparasites was not supported. Whereas homoxenous parasites, transmitted predominantly by intraspecies contact, were mainly impacted by host sociality and/or sleeping site characteristics, parasites with free‐living generations or intermediate arthropod hosts in their life cycles were all impacted by habitat conditions and showed negative responses to forest edges and/or vegetation clearance. Such negative effects probably result from negative abiotic impacts on either free‐living stages and/or intermediate hosts, although their investigation in a nonexperimental setting precludes full clarification of causality. Additionally, we found strong evidence that pristine forests with dense vegetation harbored the most diverse communities of gastrointestinal parasites. Our results provide insights into the complex relationships between gastrointestinal parasites and their environment and propose them as important indicators of habitat integrity. Parasites are suggested to provide vital ecosystem services, for example, by stabilizing a high host species diversity through controlling effects on common or invasive species which may otherwise outcompete rarer native species (Lafferty, 2003; Mouritsen & Poulin, 2005). In that sense, a high parasite diversity can be regarded as a sign of a healthy ecosystem (Hudson et al., 2006). In conclusion, this study shows that habitat fragmentation in northwestern Madagascar has negative effects on the native gastrointestinal parasite communities of native small mammals and even the invasive *R. rattus*. Further research will be needed to clarify the underlying causal effects, to evaluate the host–parasite networks in fragile fragmented environments in more depth, starting by clarifying the taxonomy and the specific life cycles of the different parasite species. Overall, the results demonstrate that forest fragmentation should not only be regarded as a threat to the diverse suite of host species that inhabit such habitats (Andriatsitohaina et al., 2020; Steffens & Lehman, 2018) but also to their suite of often highly adapted and coevolved parasites. Future conservation planning should take these complex evolutionary relationships and habitat requirements of native parasites into account, since they may be even more vulnerable than their hosts.

## CONFLICT OF INTEREST

No conflict of interest has been declared by the authors.

## AUTHOR CONTRIBUTION

**Frederik Kiene:** Data curation (supporting); Formal analysis (lead); Investigation (lead); Methodology (equal); Validation (equal); Visualization (lead); Writing‐original draft (lead); Writing‐review & editing (equal). **Bertrand Andriatsitohaina:** Investigation (supporting); Writing‐review & editing (supporting). **Malcolm S. Ramsay:** Investigation (supporting); Writing‐review & editing (supporting). **Romule Rakotondravony:** Investigation (supporting); Methodology (supporting); Project administration (supporting); Supervision (supporting); Writing‐review & editing (supporting). **Christina Strube:** Conceptualization (equal); Data curation (supporting); Formal analysis (supporting); Methodology (supporting); Resources (supporting); Supervision (equal); Validation (equal); Writing‐review & editing (supporting). **Ute Radespiel:** Conceptualization (equal); Data curation (equal); Formal analysis (supporting); Funding acquisition (lead); Methodology (supporting); Project administration (lead); Resources (lead); Supervision (equal); Validation (equal); Writing‐review & editing (supporting).

## PERMITS

This study received permission of the Malagasy government and Madagascar National Parks (research permit nos.: N°80/17/MEF/SG/DGF/DSAP/SCB. Rc, N°151/17/MEF/SG/DGF/DSAP/ SCB. Rc, N°84/18/MEF/ SG/DGF/DSAP/SCB. Rc., N°93/18/MEF/SG/DGF/DSAP/SCB. Rc).

## Supporting information

File S1

File S2

File S3

File S4

## Data Availability

The modeling data are publicly accessible in the Zenodo repository (https://doi.org/10.5281/zenodo.4297519) and the newly generated parasite gene sequences were deposited in GenBank under accession nos. MW520838–MW520847, MW520852, and MW520853.
