# Supplementary figures and images for: Habitat fragmentation and vegetation structure impact gastrointestinal parasites of small mammalian hosts in Madagascar
Source: Ecol Evol. 2021 May 1;11(11):6766–88. doi: 10.1002/ece3.7526 (PMC8207415; doi:10.1002/ece3.7526)

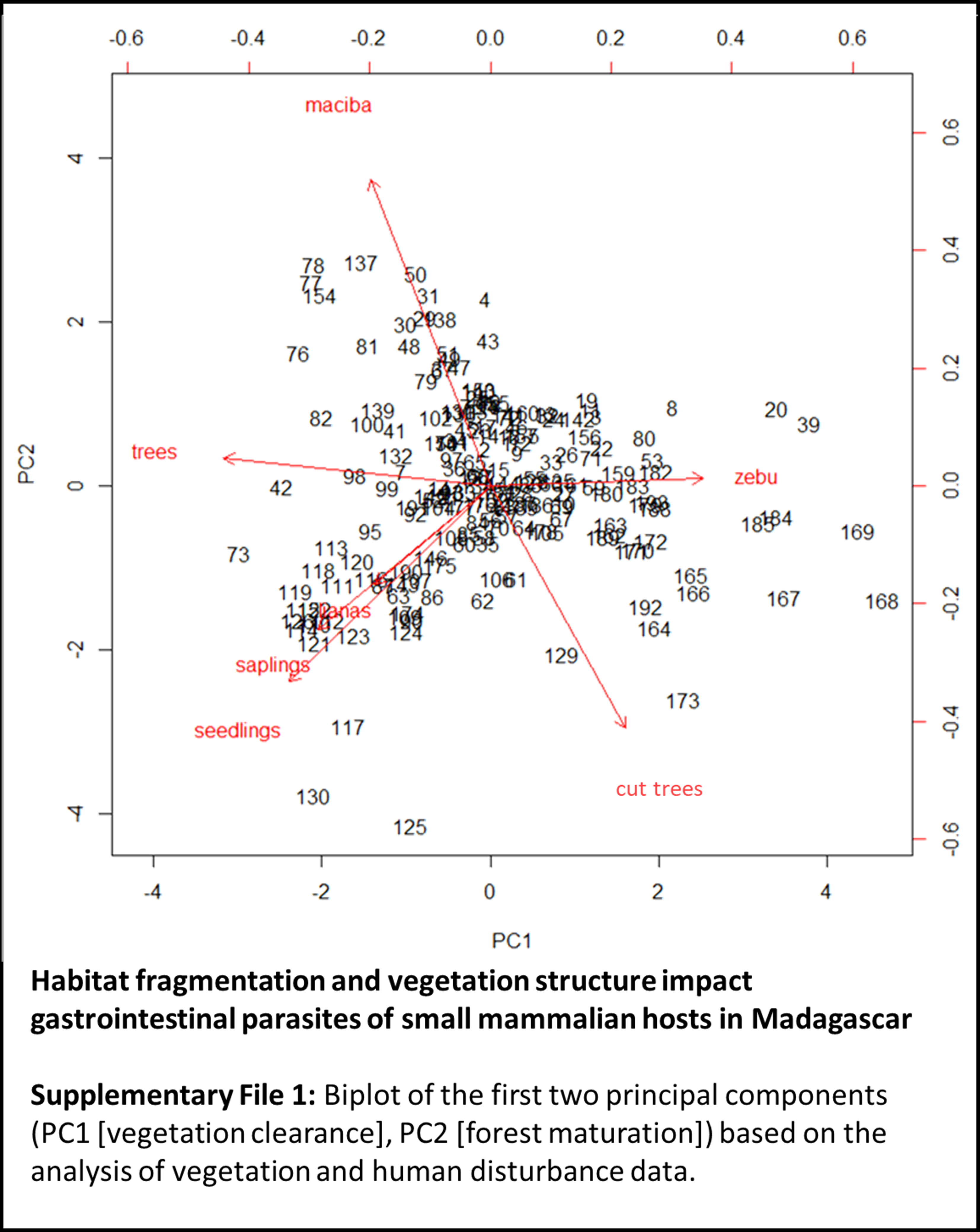

Supplement: Supplementary file 1 — File S1 [file ECE3-11-6766-s004.tif]
