# Supplementary material for: Habitat fragmentation and vegetation structure impact gastrointestinal parasites of small mammalian hosts in Madagascar
Source: Ecol Evol. 2021 May 1;11(11):6766–88. doi: 10.1002/ece3.7526 (PMC8207415; doi:10.1002/ece3.7526)
Supplement: Supplementary file 2 — File S2 [file ECE3-11-6766-s003.pdf]

# Habitat fragmentation and vegetation structure impact gastrointestinal parasites of small mammalian hosts in Madagascar

**Supplementary File 2:** Additional information on parasite species identification including detailed results of ITS1–5.8S–ITS2 sequence comparison by Geneious Alignments and BLAST comparison.

## Methods

The ITS1–5.8S–ITS2 sequence was amplified using primers NC5 and NC2 (Gasser et al. 1996) following the protocol of Klein et al. (2019). PCR-products were subsequently custom sequenced (Seqlab Sequence Laboratories Göttingen, Germany). Resulting sequences were aligned using the Geneious 10.2.3 software (https://www.geneious.com). BLAST sequence comparison was used for best possible parasite identification. Newly generated sequences were deposited in GenBank under accession nos. MW520838 - MW520847, MW520852 and MW520853.

## Results

*Parasites with a homoxenous life cycle*

### Oxyuridae

Enterobiinae gen. sp.

ITS-sequences of the first morphotype, Enterobiinae gen. sp., could be obtained for one egg sample from *M. ravelobensis* and eight excreted adult nematodes (each four from *M. murinus* and *M. ravelobensis*). BLAST comparisons showed highest identities (77-78%) to *Enterobius*

*vermicularis* (HQ646164; query cover: 39-53%). Intra-species alignments showed identities of 96-100% for *M. ravelobensis* and 100% for *M. murinus*, while inter-species identity was 98%. Morphological and genetic results indicate allocation to the Enterobiinae subfamily.

*Lemuricola* sp.

One egg sample from *M. murinus* was successfully sequenced and showed highest identities with the oxyurids *Heteroxynema cucullatum* (MH011304; identity: 73%; query cover: 74%) and *Aspiculuris tetraptera* (KY484154; identity: 91%; query cover: 42%). Unfortunately, *Lemuricola* spp. sequences are not publicly available to confirm the morphological diagnosis.

*Syphacia* sp.

While insufficient egg quantities hampered molecular analysis, two excreted adult worms containing respective eggs were successfully sequenced and showed 93% identity to the BLAST top hit *Syphacia muris* (EF464553; query cover: 99%) and was therefore assigned to that genus but an unknown species (*Syphacia* sp.).

**Strongylida**

Strongyle egg 1-3

Due to the sporadic appearance of these eggs, DNA sequencing could not be done and identification was based on morphology alone.

**Ascarididae**

Ascarid egg 1 and 2

It is very likely that the two ascarid egg morphotypes belong to the same species, however, genetic allocation of the latter three morphotypes was impaired by insufficient egg quantity.

**Trichosomoides**

*Trichosomoides crassicauda*

The egg morphotype was morphologically identified as *Trichosomoides crassicauda*. Unfortunately, genetic confirmation of our species designation failed due to insufficient availability of eggs.

**Protozoa**

Eimeriidae gen sp.

The taxonomical allocation was only performed morphologically, since the small amount of material was not sufficient for genetic characterization.

*Homoxenous parasites with homogonic or heterogonic free-living development*

*Strongyloides*

Unfortunately, molecular confirmation failed due to the low concentration of DNA yielded by processing the material. Identification is therefore based only on morphology.

*Parasites with a heteroxenous life cycle*

**Subuluroidea**

Subuluroidea fam. gen. spp.

ITS-sequences were obtained from egg samples of all host species (*M. murinus:* n = 1, *M. ravelobensis*: n = 1, *E. myoxinus*: n = 1, *R. rattus*: n = 2), and additionally from excreted adult worms (*M. murinus*: n = 1, *M. ravelobensis*: n = 5, *R. rattus*: n = 3). BLAST search showed highest identities (74-81%) with *Subulura chinensis* (MK770145-MK770149; query cover: 77-94%). Within the respective host species, sequences showed identities of more than 99%. Between the two mouse lemurs, sequences were 98% identical, while identity between the two rodents was only 68%. Similarly, identities between mouse lemurs and rodents amounted only to 68-70%. Since intraspecific ITS sequence variation is described to range around 1% (Blouin 2002), molecular results suggest the presence of three different species of the Subuluroidea superfamily. Whereas the morphotypes infecting mouse lemurs most likely belong to the same species, the two rodents probably harbored even different genera.

Subuluroidea-like egg

A genetic species determination of this morphotype was precluded by insufficient egg quantity.

**Spiruromorpha**

Spirurid egg 1

Sequencing was possible for one egg sample and four adult worms (obtained from the stomach of two individuals which accidentally died during anesthesia) from *R. rattus*. BLAST comparison of the egg sequence showed highest identities to *Mastophorus muris* (74%; MG818763; query cover: 54%), *Gongylonema nepalensis* (72%; LC388748; query cover: 52%) and *Protospirura* sp. (95%; JF514769; query cover: 28%). Three adult worms were

morphologically identified as *Protospirura muricola* and one as *Gongylonema neoplasticum*. Sequences of the three adult worms with *P. muricola* morphology showed 79-88% identity among each other, and 79-98% to the egg sequence. Two of them, gravid females, contained similar eggs as those found during coproscopy. The specimen with highest identity (98%) can be considered to belong to the same species as the sequenced eggs. However, specimens with 79% and 87% identity to the egg sequence, not differing in morphology, might suggest the presence of three cryptic species. The molecular confirmation of species affiliation to *Protospirura muricola* was, however, not possible, since sequences for that taxon are not yet publicly available. BLAST comparison of the adult worm with 98% identity to the egg sequence showed highest identities to *Gongylonema nepalensis* (75%; LC388748; query cover: 48%), *Gongylonema neoplasticum* (76%; LC388748; query cover: 48%), *Mastophorus muris* (76%; MG818763; query cover: 49%) and *Protospirura* sp. (95%; JF514769; query cover: 39%). BLAST comparisons of the adult worm sequences more distant to the egg sequence showed similarly high identities to *Gongylonema nepalensis* (75-76%; LC388754; query cover: 46%) and *Mastophorus muris* (77%; MG818763; query cover: 47%). The morphological species determination of the fourth worm as *G. neoplasticum* was confirmed by BLAST gene comparison (98% identity to *Gongylonema neoplasticum*; LC331000; query cover: 100%). Sequence identity to the egg sequence and sequences of the other worms only ranged from 43-46%. *G. neoplasticum* is, however, known to produce eggs similar to the morphotype spirurid egg 1 (da Costa Cordeiro et al. 2018). Hence, morphological and genetic results show at least for *R. rattus* hosts the presence of multiple Spiruromorpha taxa responsible for the excretion of eggs of this morphotype.

Spirurid egg 2

Sequencing could not be conducted due to low egg quantities and prevalence.

**Cestodes**

*Hymenolepis* sp.

Sequencing and ecological modeling was not possible due to low egg quantities and prevalence. The identification was hence based on morphology alone.

**References**

Blouin, M. S. 2002. Molecular prospecting for cryptic species of nematodes: Mitochondrial DNA versus internal transcribed spacer. - Int. J. Parasitol. 32: 527–531.

da Costa Cordeiro, H. et al. 2018. Gongylonema Parasites of Rodents: A Key to Species and New Data on Gongylonema neoplasticum. - J. Parasitol. 104: 51–59.

Gasser, R. B. et al. 1996. Rapid delineation of closely-related filarial parasites using genetic markers in spacer rDNA. - Acta Trop. 62: 143–150.

Klein, A. et al. 2019. Differences in infection patterns of vector-borne blood-stage parasites of sympatric Malagasy primate species (*Microcebus murinus*, *M. ravelobensis*). - Int. J. Parasitol. Parasites Wildl. 10: 59–70.
