# Supplementary material for: Habitat fragmentation and vegetation structure impact gastrointestinal parasites of small mammalian hosts in Madagascar
Source: Ecol Evol. 2021 May 1;11(11):6766–88. doi: 10.1002/ece3.7526 (PMC8207415; doi:10.1002/ece3.7526)
Supplement: Supplementary file 3 — File S3 [file ECE3-11-6766-s001.pdf]

Habitat fragmentation and vegetation structure impact gastrointestinal parasites of small mammalian hosts in Madagascar

Supplementary File 3: Numbers of positive host individuals and prevalences (in brackets) of the detected gastrointestinal parasite morphotypes, mean gastrointestinal parasite species richness (GPSR) (σ: standard deviation) and number of different parasite morphotypes detected in the different host species (morphotype repertoire).

	M. murinus ***n* = 199**	***M. ravelobensis*** ***n* = 421**	***E. myoxinus*** ***n* = 102**	***R. rattus*** ***n* = 181**	**total** ***n* = 903**
**parasites with a homoxenous life cycle (one host per life cycle)**					
Enterobiinae gen. sp.	12 (6.0%)	38 (9.0%)		1 (0.6%)	51 (5.7%)
Lemuricola sp.	32 (16.1%)	18 (4.3%)		1 (0.6%)	51 (5.7%)
Syphacia sp.				9 (5.0%)	9 (1.0%)
strongyle egg 1	1 (0.5%)	4 (1.0%)			5 (0.6%)
strongyle egg 2		6 (1.4%)		1 (0.6%)	7 (0.8%)
strongyle egg 3				3 (1.7%)	3 (0.3%)
ascarid egg 1	1 (0.5%)	3 (0.7%)			4 (0.4%)
ascarid egg 2		2 (0.45%)			2 (0.2%)
Trichosomoides crassicauda				25 (13.8%)	25 (2.8%)
Eimeriidae gen. sp.		12 (2.9%)			12 (1.3%)
**homoxenous parasites with homogonic or heterogonic free-living development (one host per life cycle, but parasites can undergo free-living generations between parasitic generations)**					
Strongyloides spp.	14 (7.0%)	170 (40.4%)		2 (1.1%)	186 (20.6%)
**parasites with a heteroxenous life cycle (intermediate host required)**					
Subuluroidea fam. gen. spp.	91 (45.7%)	226 (53.7%)	9 (8.8%)	40 (22.1%)	366 (40.5%)
Subuloroidea-like egg			1 (1.0%)	4 (2.2%)	5 (0.6%)
spirurid egg 1	16 (8.0%)	37 (8.8%)	10 (9.8%)	90 (49.7%)	153 (16.9%)
spirurid egg 2				21 (11.6%)	21 (2.3%)
Hymenolepis sp.	12 (6.0%)	15 (3.6%)			27 (3.0%)
no parasite stages detected	78 (39.2%)	105 (24.9%)	85 (83.3%)	72 (39.8%)	340 (37.7%)
mean GPSR	0.899 (σ = 0.948)	1.261 (σ = 1.123)	0.196 (σ = 0.443)	1.093 (σ = 1.045)	1.027 (σ = 1.013)
morphotype repertoire	8	11	3	11	16
