# Supplementary material for: Habitat fragmentation and vegetation structure impact gastrointestinal parasites of small mammalian hosts in Madagascar
Source: Ecol Evol. 2021 May 1;11(11):6766–88. doi: 10.1002/ece3.7526 (PMC8207415; doi:10.1002/ece3.7526)
Supplement: Supplementary file 4 — File S4 [file ECE3-11-6766-s002.pdf]

Habitat fragmentation and vegetation structure impact gastrointestinal parasites of small mammalian hosts in Madagascar

Supplementary File 4: Results for sub-models with ΔAICc < 2, used for interpretation: number of fixed factors inserted (k), degrees of freedom (df), LogLikelihood (logLik), corrected Akaike information criterion (AICc), difference of AICc to the AICc of best model (ΔAICc), Akaike weight (ω), Akaike information criterion (AIC) and estimates and *P*-values (significant values printed in bold) for each fixed factor in generalized linear mixed models calculated for each global model on each parasite morphotype and gastrointestinal parasite species richness (GSPR). Results for best models (lowest AICc) are detailed. Abbreviations: cont. = continuous forest; frag. = fragmented forest; Mm = *M. murinus*; Mr = *M. ravelobensis*; Em = *E. myoxinus*; Rr = *R. rattus*

Enterobiinae gen. spp. Model A full model: ~ sex + species + body condition + host species*cont. vs. frag. + cont. vs. frag. + vegetation clearance

								sex		species		body condition		host*cont. vs. frag.		forest category		vegetation clearance	
	**k**	**df**	**logLik**	**AICc**	**ΔAICc**	**ω**	**AIC**	**Estimate**	***P*-value**	**Estimate**	***P*-value**	**Estimate**	***P*-value**	**Estimate**	***P*-value**	**Estimate**	***P*-value**	**Estimate**	***P*-value**
**models with ΔAICc < 2**	2	8	-168.65	353.50	0.00	0.34	353.30	-0.48	0.12	**significant diff.**									
	1	7	-169.88	353.90	0.42	0.27	353.80			**significant diff.**									
	3	9	-168.55	355.30	1.84	0.13	355.10	-0.47	0.13	**significant diff.**								-0.06	0.66
	3	9	-168.56	355.30	1.86	0.13	355.10	-0.48	0.12	**significant diff.**		0.26	0.65						
	3	9	-168.59	355.40	1.91	0.13	355.20	-0.47	0.13	**significant diff.**						-0.14	0.71		

	**k**	**df**	**logLik**	**AICc**	**ΔAICc**	**ω**	**AIC**	**factor**	**Estimate**	**Std. Error**	**z value**	***P*-value**	**Effect on parasite**
**best model**	2	8	-168.65	353.50	0.00	0.34	353.30	**intercept**	-20.58	2975.73	-0.01	0.99	
								**sex**	-0.48	0.31	-1.55	0.12	
								**Mm *vs*.Em**	17.84	2975.73	0.01	1.00	
								**Rr *vs*. Em**	15.62	2975.73	0.01	1.00	
								**Mr *vs*. Em**	18.42	2975.73	0.01	1.00	
								**Rr *vs*. Mm**	-2.23	1.06	-2.10	0.11	
								**Mr *vs*. Mm**	0.58	0.37	1.55	0.34	
								**Mr *vs*. Rr**	**2.80**	**1.02**	**2.74**	**0.02**	*M. ravelobensis* > *R. rattus*

Enterobiinae gen. spp. Model B full model: ~ sex + species + body condition + host species*cont. vs. frag. + cont. vs. frag. + forest size + forest maturation

								sex		species		body condition		host*cont. vs. frag.		forest category		forest size		forest maturation	
	**k**	**df**	**logLik**	**AICc**	**ΔAICc**	**ω**	**AIC**	**Estimate**	***P*-value**	**Estimate**	***P*-value**	**Estimate**	***P*-value**	**Estimate**	***P*-value**	**Estimate**	***P*-value**	**Estimate**	***P*-value**	**Estimate**	***P*-value**
**models with ΔAICc < 2**	3	9	-166.05	350.30	0.00	0.52	350.10	-0.56	0.07	**significant diff.**										**-0.39**	**0.02**
	2	8	-167.68	351.50	1.21	0.28	351.40			**significant diff.**										**-0.36**	**0.03**
	4	10	-165.97	352.20	1.89	0.20	351.90	-0.55	0.08	not significant								0.02	0.68	**-0.39**	**0.02**

	**k**	**df**	**logLik**	**AICc**	**ΔAICc**	**ω**	**AIC**	**factor**	**Estimate**	**Std. Error**	**z value**	***P*-value**	**Effect on parasite**
**best model**	3	9	-166.05	350.30	0.00	0.52	350.10	**intercept**	-20.62	92.71	-0.22	0.82	
								**sex**	-0.56	0.31	-1.79	0.07	
								**Mm *vs*.Em**	17.94	92.71	0.19	0.10	
								**Rr *vs*. Em**	15.69	92.71	0.17	0.10	
								**Mr *vs*. Em**	18.20	92.71	0.20	0.10	
								**Rr *vs*. Mm**	-2.25	1.05	-2.14	0.11	
								**Mr *vs*. Mm**	0.26	0.40	0.66	0.89	
								**Mr *vs*. Rr**	**2.51**	**1.02**	**2.46**	**0.05**	*M. ravelobensis* > *R. rattus*
								**forest maturation**	**-0.39**	**0.17**	**-2.30**	**0.02**	less parasites in hosts from mature and pristine habitats

Enterobiinae gen. spp. Model C full model: ~ sex + species + density + species*cont. vs. frag. + cont. vs. frag. + distance to edge

								sex		species		density		host*cont. vs. frag.		forest category		dist. edge	
	**k**	**df**	**logLik**	**AICc**	**ΔAICc**	**ω**	**AIC**	**Estimate**	***P*-value**	**Estimate**	***P*-value**	**Estimate**	***P*-value**	**Estimate**	***P*-value**	**Estimate**	***P*-value**	**Estimate**	***P*-value**
**models with ΔAICc < 2**	1	7	-177.06	368.30	0.00	0.45	368.10			**significant diff.**									
	2	8	-176.37	368.90	0.60	0.33	368.70	-0.35	0.24	**significant diff.**									
	2	8	-176.76	369.70	1.40	0.22	369.50			**significant diff.**								0.10	0.44

	**k**	**df**	**logLik**	**AICc**	**ΔAICc**	**ω**	**AIC**	**factor**	**Estimate**	**Std. Error**	**z value**	***P*-value**	**Effect on parasite**
**best model**	1	7	-177.06	368.30	0.00	0.45	368.10	**intercept**	-20.76	87.81	-0.24	0.81	
								**Mm *vs*.Em**	17.86	87.81	0.20	1.00	
								**Rr *vs*. Em**	15.44	87.81	0.18	1.00	
								**Mr *vs*. Em**	18.34	87.81	0.21	1.00	
								**Rr *vs*. Mm**	-2.42	1.04	-2.33	0.07	
								**Mr *vs*. Mm**	0.48	0.37	1.30	0.50	
								**Mr *vs*. Rr**	**2.90**	**1.01**	**2.86**	**0.01**	*M. ravelobensis* > *R. rattus*

Enterobiinae gen. spp. Model D full model: ~ sex + species + density + edge percentage

	k	df	logLik	AICc	ΔAICc	ω	AIC	sex Estimate	sex *P*-value	species Estimate	species *P*-value	density Estimate	density *P*-value	edge percentage Estimate	edge percentage *P*-value
models with ΔAICc < 2	2	8	-64.93	146.20	0.00	0.30	145.90			**significant diff.**				-2.12	0.11
	1	7	-66.24	146.70	0.60	0.23	146.50			**significant diff.**					
	3	9	-64.21	146.80	0.50	0.22	146.40	-0.61	0.24	**significant diff.**				-2.20	0.09
	2	8	-65.68	147.70	1.50	0.14	147.40	-0.53	0.29	**significant diff.**					
	3	9	-64.88	148.20	1.90	0.11	147.80			**significant diff.**		0.11	0.77	-2.21	0.12

	k	df	logLik	AICc	ΔAICc	ω	AIC	factor	Estimate	Std. Error	z value	*P*-value	Effect on parasite
best model	2	8	-64.93	146.20	0.00	0.30	145.90	**intercept**	-20.34	142.00	-0.14	0.89	
								**Mm *vs* .Em**	18.93	142.01	0.13	1.00	
								**Rr *vs* . Em**	16.77	142.01	0.12	1.00	
								**Mr *vs* . Em**	19.46	142.00	0.14	1.00	
								**Rr *vs* . Mm**	-2.16	1.07	-2.01	0.14	
								**Mr *vs* . Mm**	0.53	0.58	0.92	0.75	
								**Mr *vs* . Rr**	**2.69**	**1.08**	**2.49**	**0.04**	*M. ravelobensis* > *R. rattus*
								**edgepercent**	-2.12	1.39	-1.53	0.11	

Enterobiinae gen. spp. Model E full model: ~ sex + species + body condition + forest area + vegetation clearance

	k	df	logLik	AICc	ΔAICc	ω	AIC	sex Estimate	sex *P*-value	species Estimate	species *P*-value	body condition Estimate	body condition *P*-value	forest size Estimate	forest size *P*-value	vegetation clearance Estimate	vegetation clearance *P*-value
models with ΔAICc < 2	2	8	-58.03	132.40	0.00	0.35	132.10	-0.93	0.10	**significant diff.**							
	1	7	-59.50	133.30	0.87	0.23	133.00			**significant diff.**							
	3	9	-57.82	134.10	1.67	0.15	133.60	-0.94	0.09	**significant diff.**		0.58	0.48				
	3	9	-57.93	134.30	1.90	0.14	133.90	-0.89	0.12	**significant diff.**				-0.10	0.66		
	3	9	-57.95	134.30	1.93	0.13	133.90	-0.95	0.09	**significant diff.**						0.08	0.69

	k	df	logLik	AICc	ΔAICc	ω	AIC	factor	Estimate	Std. Error	z value	*P*-value	Effect on parasite
best model	2	8	-58.03	132.40	0.00	0.35	132.10	**intercept**	-21.29	220.84	-0.10	0.92	
								**sex**	-0.93	0.56	-1.66	0.10	
								**Mm *vs* .Em**	18.48	220.84	0.08	1.00	
								**Rr *vs* . Em**	16.80	220.84	0.08	1.00	
								**Mr *vs* . Em**	19.59	220.84	0.09	1.00	
								**Rr *vs* . Mm**	-1.68	1.09	-1.55	0.35	
								**Mr *vs* . Mm**	1.11	0.55	2.02	0.14	
								**Mr *vs* . Rr**	**2.80**	**1.07**	**2.62**	**0.03**	*M. ravelobensis* > *R. rattus*

Enterobiinae gen. spp. Model F full model: ~ sex + species + distance to edge + forest maturation

	k	df	logLik	AICc	ΔAICc	ω	AIC	sex Estimate	sex *P*-value	species Estimate	species *P*-value	dist. edge Estimate	dist. edge *P*-value	forest maturation Estimate	forest maturation *P*-value
models with ΔAICc < 2	2	8	-58.03	132.40	0.00	0.40	132.10	-0.93	0.10	**significant diff.**					
	1	7	-59.50	133.30	0.87	0.26	133.00			**significant diff.**					
	3	9	-57.73	133.90	1.50	0.19	133.50	-0.94	0.10	**significant diff.**		0.28	0.45		
	3	9	-57.96	134.40	1.96	0.15	133.90	-0.91	0.11	**significant diff.**				0.10	0.72

	k	df	logLik	AICc	ΔAICc	ω	AIC	factor	Estimate	Std. Error	z value	*P*-value	Effect on parasite
best model	2	8	-58.03	132.40	0.00	0.40	132.10	**intercept**	-21.29	220.84	-0.10	0.92	
								**sex**	-0.93	0.56	-1.66	0.10	
								**Mm *vs* .Em**	18.48	220.84	0.08	1.00	
								**Rr *vs* . Em**	16.80	220.84	0.08	1.00	
								**Mr *vs* . Em**	19.59	220.84	0.09	1.00	
								**Rr *vs* . Mm**	-1.68	1.09	-1.55	0.35	
								**Mr *vs* . Mm**	1.11	0.55	2.02	0.14	
								**Mr *vs* . Rr**	**2.80**	**1.07**	**2.62**	**0.03**	*M. ravelobensis* > *R. rattus*

***Lemuricola* sp. Model A** full model: ~ sex + species + body condition + host species*cont. vs. frag. + cont. vs. frag. + vegetation clearance

	k	df	logLik	AICc	ΔAICc	ω	AIC	sex		species		body condition		host*cont. vs. frag.		forest category		vegetation clearance	
								Estimate	*P*-value	Estimate	*P*-value	Estimate	*P*-value	Estimate	*P*-value	Estimate	*P*-value	Estimate	*P*-value
models with ΔAICc < 2	3	9	-142.83	303.90	0.00	0.26	303.70	0.53	0.13	significant diff.		0.85	0.08						
	2	8	-143.88	303.90	0.06	0.25	303.80	0.56	0.10	significant diff.									
	2	8	-144.02	304.20	0.34	0.22	304.00			significant diff.		0.87	0.06						
	1	7	-145.21	304.60	0.69	0.18	304.40			significant diff.									
	4	10	-142.80	305.90	1.99	0.10	305.60	0.52	0.13	significant diff.		0.86	0.08			0.11	0.81		

	k	df	logLik	AICc	ΔAICc	ω	AIC	factor	Estimate	Std. Error	z value	*P*-value	Effect on parasite
best model	3	9	-142.83	303.90	0.00	0.26	303.70	intercept	-21.33	1677.75	-0.01	0.99	
								sex	0.53	0.35	1.53	0.13	
								Mm *vs*.Em	17.73	1677.75	0.01	1.00	
								Rr *vs*. Em	14.08	1677.75	0.01	1.00	
								Mr *vs*. Em	16.75	1677.75	0.01	1.00	
								Rr *vs*. Mm	**-3.65**	**1.10**	**-3.32**	**< 0.001**	*M. murinus* > *R. rattus*
								Mr *vs*. Mm	**-0.98**	**0.39**	**-2.50**	**0.04**	*M. murinus* > *M. ravelobensis*
								Mr *vs*. Rr	2.68	1.12	2.39	0.06	
								body condition	0.85	0.48	1.77	0.08	

***Lemuricola* sp. Model B** full model: ~ sex + species + body condition + host species*cont. vs. frag. + cont. vs. frag. + forest size + forest maturation

	k	df	logLik	AICc	ΔAICc	ω	AIC	sex		species		body condition		host*cont. vs. frag.		forest category		forest size		forest maturation	
								Estimate	*P*-value	Estimate	*P*-value	Estimate	*P*-value	Estimate	*P*-value	Estimate	*P*-value	Estimate	*P*-value	Estimate	*P*-value
models with ΔAICc < 2	4	10	-140.89	302.00	0.00	0.29	301.80	0.68	0.06	significant diff.		0.91	0.06							**0.46**	**0.04**
	3	9	-142.08	302.40	0.34	0.24	302.20	**0.71**	**0.05**	significant diff.										**0.45**	**0.04**
	3	9	-142.74	303.70	1.66	0.13	303.50			significant diff.		**0.93**	**0.04**							0.36	0.08
	3	9	-142.83	303.90	1.84	0.12	303.70	0.53	0.13	significant diff.		0.85	0.08								
	2	8	-143.88	303.90	1.89	0.11	303.80	0.56	0.10	significant diff.											
	5	11	-140.81	303.90	1.90	0.11	303.60	0.67	0.06	significant diff.		0.92	0.06					-0.02	0.70	**0.46**	**0.04**

	k	df	logLik	AICc	ΔAICc	ω	AIC	factor	Estimate	Std. Error	z value	*P*-value	Effect on parasite
best model	4	10	-140.89	302.00	0.00	0.29	301.80	intercept	-21.44	1696.27	-0.01	0.99	
								sex	0.68	0.36	1.89	0.06	
								Mm *vs*.Em	17.76	1696.27	0.01	1.00	
								Rr *vs*. Em	14.05	1696.27	0.01	1.00	
								Mr *vs*. Em	16.91	1696.27	0.01	1.00	
								Rr *vs*. Mm	**-3.71**	**1.11**	**-3.34**	**< 0.001**	*M. murinus* > *R. rattus*
								Mr *vs*. Mm	-0.85	0.39	-2.19	0.09	
								Mr *vs*. Rr	**2.86**	**1.13**	**2.52**	**0.04**	*M. ravelobensis* > *R. rattus*
								body condition	0.91	0.49	1.89	0.06	
								forest maturation	**0.46**	**0.22**	**2.09**	**0.04**	more parasites in hosts from mature and pristine habitats

***Lemuricola* sp. Model C** full model: ~ sex + species + density + species*cont. vs. frag. + cont. vs. frag. + distance to edge

	k	df	logLik	AICc	ΔAICc	ω	AIC	sex		species		density		host*cont. vs. frag.		forest category		dist. edge	
								Estimate	*P*-value	Estimate	*P*-value	Estimate	*P*-value	Estimate	*P*-value	Estimate	*P*-value	Estimate	*P*-value
models with ΔAICc < 2	2	8	-153.58	323.30	0.00	0.37	323.20	**0.66**	**0.04**	significant diff.									
	3	9	-153.10	324.40	1.00	0.21	324.20	0.65	0.05	significant diff.								-0.13	0.32
	3	9	-153.50	325.20	1.80	0.14	325.00	**0.65**	**0.05**	significant diff.						0.17	0.68		
	3	9	-153.52	325.20	1.80	0.14	325.00	0.65	0.05	significant diff.		0.10	0.73						
	1	7	-155.58	325.30	2.00	0.14	325.20			significant diff.									

	k	df	logLik	AICc	ΔAICc	ω	AIC	factor	Estimate	Std. Error	z value	*P*-value	Effect on parasite
best model	2	8	-153.58	323.30	0.00	0.37	323.20	intercept	-20.60	84.75	-0.24	0.81	
								sex	**0.66**	**0.32**	**2.03**	**0.04**	more parasites in male hosts
								Mm *vs*.Em	17.98	84.75	0.21	1.00	
								Rr *vs*. Em	14.27	84.75	0.17	1.00	
								Mr *vs*. Em	16.85	84.75	0.20	1.00	
								Rr *vs*. Mm	**-3.71**	**1.01**	**-3.69**	**< 0.004**	*M. murinus* > *R. rattus*
								Mr *vs*. Mm	**-1.13**	**0.37**	**-3.05**	**0.01**	*M. murinus* > *M. ravelobensis*
								Mr *vs*. Rr	**2.58**	**1.03**	**2.51**	**0.04**	*M. ravelobensis* > *R. rattus*

Lemuricola sp. Model D full model: ~ sex + species + density + edge percentage

	k	df	logLik	AICc	ΔAICc	ω	AIC	sex Estimate	sex *P*-value	species Estimate	species *P*-value	density Estimate	density *P*-value	edge percentage Estimate	edge percentage *P*-value
models with ΔAICc < 2	1	7	-87.47	189.20	0.00	0.42	188.90			not significant					
	2	8	-86.64	189.60	0.40	0.34	189.30	0.56	0.20	not significant					
	2	8	-87.01	190.30	1.10	0.24	190.00			not significant		0.37	0.32		

	k	df	logLik	AICc	ΔAICc	ω	AIC	factor	Estimate	Std. Error	z value	*P*-value	Effect on parasite
best model	1	7	-87.47	189.20	0.00	0.42	188.90	**intercept**	-30.74	173.40	-0.18	0.86	
								**Mm *vs* .Em**	28.27	173.40	0.16	1.00	
								**Rr *vs* . Em**	28.27	173.40	0.16	1.00	
								**Mr *vs* . Em**	28.27	173.40	0.16	1.00	
								**Rr *vs* . Mm**	-37.70	725.37	-0.05	1.00	
								**Mr *vs* . Mm**	-0.68	0.48	-1.44	0.40	
								**Mr *vs* . Rr**	37.02	725.37	0.05	1.00	

Lemuricola sp. Model E full model: ~ sex + species + body condition + forest area + vegetation clearance

	k	df	logLik	AICc	ΔAICc	ω	AIC	sex Estimate	sex *P*-value	species Estimate	species *P*-value	body condition Estimate	body condition *P*-value	forest size Estimate	forest size *P*-value	vegetation clearance Estimate	vegetation clearance *P*-value
models with ΔAICc < 2	2	8	-78.10	172.50	0.00	0.25	172.20			not significant				-0.34	0.15		
	1	7	-79.16	172.60	0.05	0.24	172.30			not significant							
	2	8	-78.68	173.70	1.16	0.14	173.40	0.45	0.33	not significant							
	3	9	-77.65	173.70	1.18	0.14	173.30	0.44	0.35	not significant				-0.33	0.16		
	3	9	-77.78	174.00	1.44	0.12	173.60			not significant				-0.35	0.13	-0.23	0.42
	2	8	-78.94	174.20	1.68	0.11	173.90			not significant						-0.19	0.50

	k	df	logLik	AICc	ΔAICc	ω	AIC	factor	Estimate	Std. Error	z value	*P*-value	Effect on parasite
best model	2	8	-78.10	172.50	0.00	0.25	172.20	**intercept**	-25.03	24800.00	0.00	1.00	
								**Mm *vs* .Em**	23.11	24800.00	0.00	1.00	
								**Rr *vs* . Em**	-2041.00	5955000.00	0.00	1.00	
								**Mr *vs* . Em**	22.94	24800.00	0.00	1.00	
								**Rr *vs* . Mm**	-2064.00	5955000.00	0.00	1.00	
								**Mr *vs* . Mm**	-0.17	0.57	-0.29	0.99	
								**Mr *vs* . Rr**	2064.00	5955000.00	0.00	1.00	
								**forest size**	-0.34	0.23	-1.45	0.15	

Lemuricola sp. Model F full model: ~ sex + species + distance to edge + forest maturation

	k	df	logLik	AICc	ΔAICc	ω	AIC	sex Estimate	sex *P*-value	species Estimate	species *P*-value	dist. edge Estimate	dist. edge *P*-value	forest maturation Estimate	forest maturation *P*-value
models with ΔAICc < 2	3	9	-75.70	169.80	0.00	0.33	169.40	0.71	0.15	not significant				**0.77**	**0.01**
	2	8	-76.81	170.00	0.13	0.31	169.60			not significant				**0.65**	**0.03**
	4	10	-75.19	170.90	1.08	0.19	170.40	0.76	0.12	not significant		0.30	0.33	**0.79**	**0.01**
	3	9	-76.44	171.30	1.47	0.16	170.90			not significant		0.26	0.40	**0.67**	**0.02**

	k	df	logLik	AICc	ΔAICc	ω	AIC	factor	Estimate	Std. Error	z value	*P*-value	Effect on parasite
best model	3	9	-75.70	169.80	0.00	0.33	169.40	**intercept**	-30.36	194100.00	0.00	1.00	
								**Mm *vs* .Em**	27.28	194100.00	0.00	1.00	
								**Rr *vs* . Em**	-97.75	5958000.00	0.00	1.00	
								**Mr *vs* . Em**	26.81	194100.00	0.00	1.00	
								**Rr *vs* . Mm**	-125.00	5955000.00	0.00	1.00	
								**Mr *vs* . Mm**	-0.47	0.51	-0.91	0.74	
								**Mr *vs* . Rr**	124.60	5955000.00	0.00	1.00	
								**sex**	0.71	0.49	1.45	0.15	
								**forest maturation**	**0.77**	**0.32**	**2.44**	**0.01**	more parasites in hosts from mature and pristine habitats

Strongyloides **spp. Model A** full model: ~ sex + species + body condition + host species*cont. vs. frag. + cont. vs. frag. + vegetation clearance

models with ΔAICc < 2

k	df	logLik	AICc	ΔAICc	ω	AIC	sex Estimate	sex *P*-value	species Estimate	species *P*-value	body condition Estimate	body condition *P*-value	host*cont. vs. frag. Estimate	host*cont. vs. frag. *P*-value	forest category Estimate	forest category *P*-value	vegetation clearance Estimate	vegetation clearance *P*-value
3	9	-312.18	642.60	0.00	0.48	642.40			**significant diff.**						**-0.92**	**0.01**	**-0.23**	**0.04**
4	10	-344.60	643.50	0.90	0.30	643.20			**significant diff.**		0.44	0.25			**-0.91**	**0.01**	**-0.24**	**0.03**
4	10	-311.90	644.10	1.51	0.22	643.80	-0.15	0.46	**significant diff.**						**-0.92**	**0.01**	**-0.23**	**0.04**

best model

k	df	logLik	AICc	ΔAICc	ω	AIC	factor	Estimate	Std. Error	z value	*P*-value	Effect on parasite
3	9	-312.18	642.60	0.00	0.48	642.40	**intercept**	-17.97	1113.58	-0.02	0.99	
							**Mm *vs* .Em**	16.16	1113.58	0.02	1.00	
							**Rr *vs* . Em**	14.24	1113.58	0.01	1.00	
							**Mr *vs* . Em**	17.58	1113.58	0.02	1.00	
							**Rr *vs* . Mm**	**-1.92**	**0.79**	**-2.44**	**0.05**	*M. murinus > R. rattus*
							**Mr *vs* . Mm**	**1.41**	**0.36**	**3.90**	**<0.001**	*M. ravelobensis > M. murinus*
							**Mr *vs* . Rr**	**3.34**	**0.75**	**4.44**	**<0.001**	*M. ravelobensis > R. rattus*
							**forest category**	**-0.92**	**0.34**	**-2.73**	**0.01**	more parasites on hosts from the continuous forest
							**vegetation clearance**	**-0.23**	**0.11**	**-2.03**	**0.04**	less parasites in hosts from habitats with low vegetation density

Strongyloides **spp. Model B** full model: ~ sex + species + body condition + host species*cont. vs. frag. + cont. vs. frag. + forest size + forest maturation

models with ΔAICc < 2

k	df	logLik	AICc	ΔAICc	ω	AIC	sex Estimate	sex *P*-value	species Estimate	species *P*-value	body condition Estimate	body condition *P*-value	host*cont. vs. frag. Estimate	host*cont. vs. frag. *P*-value	forest category Estimate	forest category *P*-value	forest size Estimate	forest size *P*-value	forest maturation Estimate	forest maturation *P*-value
3	9	-312.51	643.20	0.00	0.40	643.00			**significant diff.**						**-2.35**	**<0.001**	-0.18	0.06		
4	10	-312.07	644.40	1.18	0.22	644.10			**significant diff.**		0.38	0.32			**-2.36**	**<0.001**	-0.18	0.06		
2	8	-314.24	644.70	1.42	0.20	644.50			**significant diff.**						**-1.28**	**<0.001**				
4	10	-312.28	644.80	1.59	0.18	644.60	-0.14	0.50	**significant diff.**						**-2.33**	**<0.001**	-0.18	0.07		

best model

k	df	logLik	AICc	ΔAICc	ω	AIC	factor	Estimate	Std. Error	z value	*P*-value	Effect on parasite
3	9	-312.51	643.20	0.00	0.40	643.00	**intercept**	-16.12	1103.94	-0.02	0.99	
							**Mm *vs* .Em**	16.09	1103.94	0.02	1.00	
							**Rr *vs* . Em**	14.23	1103.94	0.01	1.00	
							**Mr *vs* . Em**	17.54	1103.94	0.02	1.00	
							**Rr *vs* . Mm**	-1.86	0.79	-2.37	0.06	
							**Mr *vs* . Mm**	**1.45**	**0.37**	**3.93**	**< 0.001**	*M. ravelobensis > M. murinus*
							**Mr *vs* . Rr**	**3.31**	**0.75**	**4.41**	**< 1e-04**	*M. ravelobensis > R. rattus*
							**forest category**	**-2.35**	**0.64**	**-3.67**	**< 0.001**	more parasites on hosts from the continuous forest
							**forest size**	-0.18	0.10	-1.87	0.06	

Strongyloides **spp. Model C** full model: ~ sex + species + density + species*cont. vs. frag. + cont. vs. frag. + distance to edge

models with ΔAICc < 2

k	df	logLik	AICc	ΔAICc	ω	AIC	sex Estimate	sex *P*-value	species Estimate	species *P*-value	density Estimate	density *P*-value	host*cont. vs. frag. Estimate	host*cont. vs. frag. *P*-value	forest category Estimate	forest category *P*-value	dist. edge Estimate	dist. edge *P*-value
3	9	-320.62	659.40	0.00	0.49	659.20			**significant diff.**						-0.57	0.08	**0.40**	**<0.001**
2	8	-322.11	660.40	1.00	0.31	660.20			**significant diff.**								**0.52**	**<0.001**
4	10	-320.51	661.30	1.80	0.20	661.00	-0.09	0.64	**significant diff.**						-0.56	0.09	**0.40**	**<0.001**

best model

k	df	logLik	AICc	ΔAICc	ω	AIC	factor	Estimate	Std. Error	z value	*P*-value	Effect on parasite
3	9	-320.62	659.40	0.00	0.49	659.20	**intercept**	-20.91	88.47	-0.24	0.81	
							**Mm *vs* .Em**	17.02	88.46	0.19	1.00	
							**Rr *vs* . Em**	15.11	88.46	0.17	1.00	
							**Mr *vs* . Em**	18.45	88.46	0.21	1.00	
							**Rr *vs* . Mm**	**-1.92**	**0.77**	**-2.50**	**0.04**	*M. murinus > R. rattus*
							**Mr *vs* . Mm**	**1.42**	**0.34**	**4.19**	**<0.001**	*M. ravelobensis > M. murinus*
							**Mr *vs* . Rr**	**3.34**	**0.73**	**4.55**	**<0.001**	*M. ravelobensis > R. rattus*
							**forest category**	-0.57	0.33	-1.72	0.08	
							**dist. edge**	**0.40**	**0.12**	**3.26**	**< 0.001**	hosts with higher distance of their capture place to the forest edge have more parasites

Strongyloides spp. Model D   full model: ~ sex + species + density + edge percentage

	k	df	logLik	AICc	ΔAICc	ω	AIC	sex Estimate	sex *P*-value	species Estimate	species *P*-value	density Estimate	density *P*-value	edge percentage Estimate	edge percentage *P*-value
models with ΔAICc < 2	2	8	-76.23	168.80	0.00	0.29	168.50			**significant diff.**		-0.53	0.12		
	1	7	-77.47	169.20	0.40	0.24	168.90			**significant diff.**					
	3	9	-75.48	169.50	0.70	0.20	169.20			**significant diff.**		-0.55	0.06	-1.59	0.18
	3	9	-75.97	170.30	1.40	0.13	169.90	0.33	0.47	**significant diff.**		-0.56	0.11		
	2	8	-77.03	170.40	1.60	0.13	170.10			**significant diff.**				-1.33	0.36

	k	df	logLik	AICc	ΔAICc	ω	AIC	factor	Estimate	Std. Error	z value	*P*-value	Effect on parasite
best model	2	8	-76.23	168.80	0.00	0.29	168.50	**intercept**	-21.31	92.52	-0.23	0.82	
								**Mm *vs* .Em**	19.07	92.52	0.21	1.00	
								**Rr *vs* . Em**	16.74	92.52	0.18	1.00	
								**Mr *vs* . Em**	20.95	92.52	0.23	0.99	
								**Rr *vs* . Mm**	-2.33	1.05	-2.22	0.09	
								**Mr *vs* . Mm**	**1.87**	**0.50**	**3.72**	**<0.001**	*M. ravelobensis* > *M. murinus*
								**Mr *vs* . Rr**	**4.20**	**1.07**	**3.93**	**<0.001**	*M. ravelobensis* > *R. rattus*
								**density**	-0.53	0.34	-1.56	0.12	

Strongyloides spp. Model E   full model: ~ sex + species + body condition + forest size + vegetation clearance

	k	df	logLik	AICc	ΔAICc	ω	AIC	sex Estimate	sex *P*-value	species Estimate	species *P*-value	body condition Estimate	body condition *P*-value	forest size Estimate	forest size *P*-value	vegetation clearance Estimate	vegetation clearance *P*-value
models with ΔAICc < 2	2	8	-72.03	160.40	0.00	0.39	160.10			**significant diff.**				**0.45**	**0.04**		
	1	7	-73.44	161.20	0.75	0.27	160.90			**significant diff.**							
	3	9	-71.65	161.70	1.34	0.20	161.30			**significant diff.**		0.68	0.34	0.45	0.19		
	3	9	-71.98	162.40	1.99	0.14	162.00			**significant diff.**				0.43	0.07	-0.06	0.75

	k	df	logLik	AICc	ΔAICc	ω	AIC	factor	Estimate	Std. Error	z value	*P*-value	Effect on parasite
best model	2	8	-72.03	160.40	0.00	0.39	160.10	**intercept**	-23.73	218.32	-0.11	0.91	
								**Mm *vs* .Em**	18.96	218.32	0.09	1.00	
								**Rr *vs* . Em**	16.76	218.32	0.08	1.00	
								**Mr *vs* . Em**	20.07	218.32	0.09	1.00	
								**Rr *vs* . Mm**	-2.20	1.07	-2.06	0.13	
								**Mr *vs* . Mm**	1.10	0.50	2.23	0.09	
								**Mr *vs* . Rr**	**3.31**	**1.05**	**3.15**	**0.01**	*M. ravelobensis* > *R. rattus*
								**forest size**	**0.45**	**0.22**	**2.01**	**0.04**	more parasites on host from larger forests

Strongyloides spp. Model F   full model: ~ sex + species + distance to edge + forest maturation

	k	df	logLik	AICc	ΔAICc	ω	AIC	sex Estimate	sex *P*-value	species Estimate	species *P*-value	dist. edge Estimate	dist. edge *P*-value	forest maturation Estimate	forest maturation *P*-value
models with ΔAICc < 2	2	8	-71.58	159.50	0.00	0.53	159.20			**significant diff.**		0.67	0.06		
	3	9	-71.35	161.10	1.63	0.24	160.70			**significant diff.**		**0.73**	**0.04**	0.25	0.43
	1	7	-73.44	161.20	1.65	0.23	160.90			**significant diff.**					

	k	df	logLik	AICc	ΔAICc	ω	AIC	factor	Estimate	Std. Error	z value	*P*-value	Effect on parasite
best model	2	8	-71.58	159.50	0.00	0.53	159.20	**intercept**	-25.08	5515.81	-0.01	1.00	
								**Mm *vs* .Em**	19.07	5515.80	0.00	1.00	
								**Rr *vs* . Em**	16.88	5515.81	0.00	1.00	
								**Mr *vs* . Em**	20.49	5515.80	0.00	1.00	
								**Rr *vs* . Mm**	-2.19	1.08	-2.02	0.14	
								**Mr *vs* . Mm**	**1.42**	**0.49**	**2.89**	**0.01**	*M. ravelobensis* > *M. murinus*
								**Mr *vs* . Rr**	**3.61**	**1.07**	**3.39**	**< 0.001**	*M. ravelobensis* > *R. rattus*
								**distance from edge**	0.67	0.35	1.90	0.06	

Subuluroidea fam. gen. spp. Model A   full model: ~ sex + species + body condition + host species*cont. vs. frag. + cont. vs. frag. + vegetation clearance

								sex		species		body condition		host*cont. vs. frag.		forest category		vegetation clearance	
	k	df	logLik	AICc	ΔAICc	ω	AIC	Estimate	*P* -value	Estimate	*P* -value	Estimate	*P* -value	Estimate	*P* -value	Estimate	*P* -value	Estimate	*P* -value
models with ΔAICc < 2	4	12	-471.06	966.50	0.00	0.40	966.10			significant diff.				not significant		15.57	0.86	**-0.19**	**0.05**
	5	13	-470.57	967.60	1.09	0.23	967.10			significant diff.		0.26	0.31	not significant		15.60	0.99	**-0.20**	**0.03**
	5	13	-470.65	967.70	1.24	0.21	967.30	0.15	0.36	significant diff.				not significant		15.56	0.74	**-0.19**	**0.05**
	3	11	-473.02	968.40	1.86	0.16	968.00			significant diff.				not significant		15.41	0.99		

	k	df	logLik	AICc	ΔAICc	ω	AIC	factor	Estimate	Std. Error	z value	*P* -value	Effect on parasite
best model	4	12	-471.06	966.50	0.00	0.40	966.10	**intercept**	-17.92	87.83	-0.20	0.84	
								**Mm *vs* .Em**	18.31	87.83	0.21	1.00	
								**Rr *vs* . Em**	16.37	87.82	0.19	1.00	
								**Mr *vs* . Em**	18.32	87.83	0.21	1.00	
								**Rr *vs* . Mm**	**-1.94**	**0.66**	**-2.94**	**0.01**	*M. murinus* > *R. rattus*
								**Mr *vs* . Mm**	0.01	0.53	0.02	1.00	
								**Mr *vs* . Rr**	**1.95**	**0.49**	**3.99**	**< 0.001**	*M. ravelobensis* > *R. rattus*
								**categoryfrag:hostmur**	-16.03	87.83	-0.18	0.86	
								**categoryfrag:hostrat**	-15.53	87.82	-0.18	0.86	
								**categoryfrag:hostrav**	-16.75	87.82	-0.19	0.85	
								**forest category**	15.57	87.82	0.18	0.86	
								**vegetation clearance**	**-0.19**	**0.10**	**-1.97**	**0.05**	less parasites in hosts from habitats with low vegetation density

Subuluroidea fam. gen. spp. Model B   full model: ~ sex + species + body condition + host species*cont. vs. frag. + cont. vs. frag. + forest size + forest maturation

								sex		species		body condition		host*cont. vs. frag.		forest category		forest size		forest maturation	
	k	df	logLik	AICc	ΔAICc	ω	AIC	Estimate	*P* -value	Estimate	*P* -value	Estimate	*P* -value	Estimate	*P* -value	Estimate	*P* -value	Estimate	*P* -value	Estimate	*P* -value
models with ΔAICc < 2	3	11	-473.02	968.40	0.00	0.20	968.00			significant diff.				not significant				15.41	0.99		
	4	12	-472.01	968.40	0.03	0.19	968.00			significant diff.				not significant		16.30	0.99	0.12	0.15		
	5	13	-471.59	969.60	1.25	0.11	969.20			significant diff.				not significant		16.34	0.99	0.12	0.13	0.10	0.33
	4	12	-472.63	969.60	1.27	0.11	969.30	0.14	0.37	significant diff.				not significant		15.37	0.99				
	4	12	-472.65	969.70	1.31	0.10	969.30			significant diff.		0.22	0.38	not significant				15.42	0.99		
	5	13	-471.64	969.70	1.37	0.10	969.30	0.14	0.39	significant diff.				not significant		16.25	0.82	0.11	0.17		
	4	12	-472.69	969.80	1.40	0.10	969.40			significant diff.				not significant		15.38	0.64			0.09	0.42
	5	13	-471.67	969.80	1.42	0.10	969.30			significant diff.		0.21	0.40	not significant		16.30	0.99	0.11	0.16		

	k	df	logLik	AICc	ΔAICc	ω	AIC	factor	Estimate	Std. Error	z value	*P* -value	Effect on parasite
best model	3	11	-473.02	968.40	0.00	0.20	968.00	**intercept**	-17.84	1259.51	-0.01	0.99	
								**Mm *vs* .Em**	18.26	1259.51	0.02	1.00	
								**Rr *vs* . Em**	16.42	1259.51	0.01	1.00	
								**Mr *vs* . Em**	18.44	1259.51	0.02	1.00	
								**Rr *vs* . Mm**	**-1.85**	**0.65**	**-2.82**	**0.02**	*M. murinus* > *R. rattus*
								**Mr *vs* . Mm**	0.18	0.51	0.36	0.98	
								**Mr *vs* . Rr**	**2.03**	**0.49**	**4.14**	**< 0.001**	*M. ravelobensis* > *R. rattus*
								**categoryfrag:hostmur**	-16.07	1259.51	-0.01	0.99	
								**categoryfrag:hostrat**	-15.60	1259.51	-0.01	0.99	
								**categoryfrag:hostrav**	-16.95	1259.51	-0.01	0.99	
								**forest category**	15.41	1259.51	0.01	0.99	

Subuluroidea fam. gen. spp. Model C full model: ~ sex + species + density + species*cont. vs. frag. + cont. vs. frag. + distance to edge

models with ΔAICc < 2	k	df	logLik	AICc	ΔAICc	ω	AIC	sex Estimate	sex *P*-value	species Estimate	species *P*-value	density Estimate	density *P*-value	host*cont. vs. frag. Estimate	host*cont. vs. frag. *P*-value	forest category Estimate	forest category *P*-value	dist. edge Estimate	dist. edge *P*-value
	4	12	-491.26	1006.90	0.00	0.47	1006.50			**significant diff.**				not significant		15.82	0.82	**0.50**	**< 0.001**
	5	13	-490.58	1007.60	0.60	0.34	1007.10	0.19	0.24	**significant diff.**				not significant		15.78	0.68	**0.50**	**< 0.001**
	5	13	-491.14	1008.70	1.80	0.19	1008.30			**significant diff.**		-0.09	0.62	not significant		15.90	0.78	**0.50**	**< 0.001**

best model	k	df	logLik	AICc	ΔAICc	ω	AIC	factor	Estimate	Std. Error	z value	*P*-value	Effect on parasite
	4	12	-491.26	1006.90	0.00	0.47	1006.50	**intercept**	-20.38	69.04	-0.30	0.77	
								**Mm *vs* .Em**	17.77	69.04	0.26	0.99	
								**Rr *vs* . Em**	16.25	69.04	0.24	0.99	
								**Mr *vs* . Em**	18.20	69.04	0.26	0.99	
								**Rr *vs* . Mm**	**-1.52**	**0.60**	**-2.54**	**0.04**	*M. murinus > R. rattus*
								**Mr *vs* . Mm**	0.43	0.43	0.98	0.72	
								**Mr *vs* . Rr**	**1.94**	**0.49**	**3.95**	**< 0.001**	*M. ravelobensis > R. rattus*
								**categoryfrag:hostmur**	-15.32	69.04	-0.22	0.82	
								**categoryfrag:hostrat**	-15.26	69.04	-0.22	0.83	
								**categoryfrag:hostrav**	-16.64	69.04	-0.24	0.81	
								**forest category**	15.82	69.04	0.23	0.82	
								**distance from edge**	**0.50**	**0.09**	**5.49**	**< 0.001**	hosts with higher distance of their capture place to the forest edge have more parasites

Subuluroidea fam. gen. spp. Model D full model: ~ sex + species + density + edge percentage

models with ΔAICc < 2	k	df	logLik	AICc	ΔAICc	ω	AIC	sex Estimate	sex *P*-value	species Estimate	species *P*-value	density Estimate	density *P*-value	edge percentage Estimate	edge percentage *P*-value
	2	8	-243.32	503.00	0.00	0.56	502.60			**significant diff.**				**-2.66**	**<0.001**
	3	9	-242.51	503.40	0.40	0.45	503.00			**significant diff.**		-0.27	0.20	**-2.66**	**<0.001**

best model	k	df	logLik	AICc	ΔAICc	ω	AIC	factor	Estimate	Std. Error	z value	*P*-value	Effect on parasite
	2	8	-243.32	503.00	0.00	0.56	502.60	**intercept**	-0.89	0.69	-1.30	0.20	
								**Mm *vs* .Em**	**2.53**	**0.43**	**5.83**	**< 0.001**	*M. murinus > E. myoxinus*
								**Rr *vs* . Em**	**1.11**	**0.43**	**2.59**	**0.05**	*R. rattus > E. myoxinus*
								**Mr *vs* . Em**	**1.65**	**0.48**	**3.42**	**< 0.001**	*M. ravelobensis > E. myoxinus*
								**Rr *vs* . Mm**	**-1.41**	**0.29**	**-4.83**	**< 0.001**	*M. murinus > R. rattus*
								**Mr *vs* . Mm**	-0.88	0.35	-2.52	0.05	
								**Mr *vs* . Rr**	0.53	0.37	1.42	0.48	
								**edgepercent**	**-2.66**	**0.68**	**-3.91**	**< 0.001**	more parasites in hosts from forest fragments with a lower percentage of edge habitat

Subuluroidea fam. gen. spp. Model E full model: ~ sex + species + body condition + forest area + vegetation clearance

	k	df	logLik	AICc	ΔAICc	ω	AIC	sex		species		body condition		forest size		vegetation clearance	
								Estimate	*P*-value	Estimate	*P*-value	Estimate	*P*-value	Estimate	*P*-value	Estimate	*P*-value
models with ΔAICc < 2	3	9	-227.27	473.00	0.00	0.36	472.50			**significant diff.**				0.18	0.13	**-0.38**	**0.02**
	2	8	-228.39	473.10	0.15	0.29	472.80			**significant diff.**						**-0.41**	**0.01**
	3	9	-228.08	474.60	1.61	0.16	474.20			**significant diff.**		0.31	0.42			**-0.42**	**0.01**
	4	10	-227.04	474.60	1.62	0.16	474.10			**significant diff.**		0.27	0.48	0.17	0.15	**-0.40**	**0.01**

	k	df	logLik	AICc	ΔAICc	ω	AIC	factor	Estimate	Std. Error	z value	*P*-value	Effect on parasite
best model	3	9	-227.27	473.00	0.00	0.36	472.50	**intercept**	-2.92	0.62	-4.73	0.00	
								**Mm *vs* .Em**	**2.45**	**0.45**	**5.46**	**< 0.004**	*M. murinus > E. myoxinus*
								**Rr *vs* . Em**	0.88	0.44	2.00	0.18	
								**Mr *vs* . Em**	**1.72**	**0.49**	**3.50**	**< 0.001**	*M. ravelobensis > E. myoxinus*
								**Rr *vs* . Mm**	**-1.56**	**0.32**	**-4.91**	**< 0.004**	*M. murinus > R. rattus*
								**Mr *vs* . Mm**	-0.72	0.35	-2.09	0.15	
								**Mr *vs* . Rr**	0.84	0.39	2.18	0.13	
								**forest size**	0.18	0.12	1.50	0.13	
								**vegetation clearance**	**-0.38**	**0.16**	**-2.38**	**0.02**	less parasites in hosts from habitats with low vegetation density

Subuluroidea fam. gen. spp. Model F full model: ~ sex + species + distance to edge + forest maturation

	k	df	logLik	AICc	ΔAICc	ω	AIC	sex		species		dist. edge		forest maturation	
								Estimate	*P*-value	Estimate	*P*-value	Estimate	*P*-value	Estimate	*P*-value
models with ΔAICc < 2	2	8	-222.06	460.50	0.00	0.65	460.10			**significant diff.**		**0.72**	**<0.001**		
	3	9	-221.63	461.70	1.22	0.35	461.30			**significant diff.**		**0.74**	**<0.001**	0.17	0.35

	k	df	logLik	AICc	ΔAICc	ω	AIC	factor	Estimate	Std. Error	z value	*P*-value	Effect on parasite
best model	2	8	-222.06	460.50	0.00	0.65	460.10	**intercept**	-5.57	0.97	-5.73	0.00	
								**Mm *vs* .Em**	**2.56**	**0.45**	**5.66**	**< 0.001**	*M. murinus > E. myoxinus*
								**Rr *vs* . Em**	1.00	0.45	2.24	0.11	
								**Mr *vs* . Em**	**1.74**	**0.49**	**3.55**	**< 0.001**	*M. ravelobensis > E. myoxinus*
								**Rr *vs* . Mm**	**-1.56**	**0.32**	**-4.88**	**< 0.001**	*M. murinus > R. rattus*
								**Mr *vs* . Mm**	-0.81	0.35	-2.35	0.08	
								**Mr *vs* . Rr**	0.74	0.38	1.94	0.21	
								**distance from edge**	**0.72**	**0.18**	**4.09**	**< 0.001**	hosts with higher distance of their capture place to the forest edge have more parasites

spirurid egg 1 Model A full model: ~ sex + species + body condition + host species*cont. vs. frag. + cont. vs. frag. + vegetation clearance

								sex		species		body condition		host*cont. vs. frag.		forest category		vegetation clearance	
	**k**	**df**	**logLik**	**AICc**	**ΔAICc**	**ω**	**AIC**	**Estimate**	***P*-value**	**Estimate**	***P*-value**	**Estimate**	***P*-value**	**Estimate**	***P*-value**	**Estimate**	***P*-value**	**Estimate**	***P*-value**
**models with ΔAICc < 2**	5	13	-292.62	611.70	0.00	0.48	611.20			**significant diff.**		**0.71**	**0.02**	not significant		1.01	0.36	**-0.41**	**<0.001**
	4	10	-296.20	612.70	0.98	0.30	612.40			**significant diff.**		**0.66**	**0.02**			**0.67**	**0.04**	**-0.41**	**<0.001**
	6	14	-292.36	613.20	1.55	0.22	612.70	0.16	0.47	**significant diff.**		**0.71**	**0.02**	not significant		1.00	0.37	**-0.42**	**<0.001**

	**k**	**df**	**logLik**	**AICc**	**ΔAICc**	**ω**	**AIC**	**factor**	**Estimate**	**Std. Error**	**z value**	***P*-value**	**Effect on parasite**
**best model**	5	13	-292.62	611.70	0.00	0.48	611.20	**intercept**	-4.16	1.21	-3.43	< 0.001	
								**Mm *vs*.Em**	1.49	1.14	1.31	0.54	
								**Rr *vs*. Em**	2.13	1.11	1.92	0.20	
								**Mr *vs*. Em**	0.90	1.06	0.85	0.82	
								**Rr *vs*. Mm**	0.63	0.65	0.98	0.75	
								**Mr *vs*. Mm**	-0.60	0.57	-1.06	0.70	
								**Mr *vs*. Rr**	**-1.23**	**0.48**	**-2.59**	**0.04**	*R. rattus* > *M. ravelobensis*
								**body cond.**	**0.71**	**0.30**	**2.39**	**0.02**	more parasites in hosts with better body condition
								**categoryfrag:hostmur**	-1.84	1.25	-1.47	0.14	
								**categoryfrag:hostrat**	0.14	1.19	0.12	0.90	
								**categoryfrag:hostrav**	-0.10	1.17	-0.08	0.93	
								**forest category**	1.01	1.11	0.91	0.36	
								**vegetation clearance**	**-0.41**	**0.13**	**-3.28**	**< 0.001**	less parasites in hosts from habitats with low vegetation density

spirurid egg 1 Model B full model: ~ sex + species + body condition + host species*cont. vs. frag. + cont. vs. frag. + forest size + forest maturation

								sex		species		body condition		host*cont. vs. frag.		forest category		forest size		forest maturation	
	**k**	**df**	**logLik**	**AICc**	**ΔAICc**	**ω**	**AIC**	**Estimate**	***P*-value**	**Estimate**	***P*-value**	**Estimate**	***P*-value**	**Estimate**	***P*-value**	**Estimate**	***P*-value**	**Estimate**	***P*-value**	**Estimate**	***P*-value**
**models with ΔAICc < 2**	2	8	-301.54	619.30	0.00	0.25	619.10			**significant diff.**		**0.62**	**0.03**								
	3	9	-300.74	619.70	0.44	0.20	619.50			**significant diff.**		**0.62**	**0.03**							-0.18	0.20
	4	12	-397.99	620.40	1.11	0.14	620.00			**significant diff.**		**0.67**	**0.02**	not significant		0.66	0.55				
	5	13	-297.22	620.90	1.63	0.11	620.40			**significant diff.**		**0.67**	**0.02**	not significant		0.73	0.51			-0.17	0.21
	3	9	-301.37	620.90	1.69	0.11	620.70			**significant diff.**		**0.63**	**0.03**					-0.02	0.55		
	3	9	-301.40	621.00	1.77	0.10	620.80			**significant diff.**		**0.63**	**0.03**			0.15	0.59				
	3	9	-301.43	621.10	1.82	0.10	620.90	0.10	0.63	**significant diff.**		**0.62**	**0.03**								

	**k**	**df**	**logLik**	**AICc**	**ΔAICc**	**ω**	**AIC**	**factor**	**Estimate**	**Std. Error**	**z value**	***P*-value**	**Effect on parasite**
**best model**	2	8	-301.54	619.30	0.00	0.25	619.10	**intercept**	-3.34	0.88	-3.82	< 0.001	
								**Mm *vs*.Em**	-0.07	0.45	-0.17	1.00	
								**Rr *vs*. Em**	**2.19**	**0.40**	**5.48**	**<1e-04**	*R. rattus* > *E. myoxinus*
								**Mr *vs*. Em**	0.62	0.42	1.49	0.43	
								**Rr *vs*. Mm**	**2.26**	**0.32**	**7.04**	**<1e-04**	*R. rattus* > *M. murinus*
								**Mr *vs*. Mm**	0.70	0.34	2.03	0.17	
								**Mr *vs*. Rr**	**-1.56**	**0.27**	**-5.78**	**<1e-04**	*R. rattus* > *M. ravelobensis*
								**body cond.**	**0.62**	**0.29**	**2.15**	**0.03**	more parasites in hosts with better body condition

spirurid egg 1 Model C full model: ~ sex + species + density + species*cont. vs. frag. + cont. vs. frag. + distance to edge

								sex		species		density		host*cont. vs. frag.		forest category		dist. edge	
	**k**	**df**	**logLik**	**AICc**	**ΔAICc**	**ω**	**AIC**	**Estimate**	***P*-value**	**Estimate**	***P*-value**	**Estimate**	***P*-value**	**Estimate**	***P*-value**	**Estimate**	***P*-value**	**Estimate**	***P*-value**
**models with ΔAICc < 2**	3	9	-320.95	660.10	0.00	0.18	659.90			**significant diff.**						0.52	0.09	0.21	0.05
	4	12	-317.96	660.30	0.00	0.16	659.90			**significant diff.**				not significant		1.04	0.34	0.20	0.06
	1	7	-323.33	660.80	0.80	0.13	660.70			**significant diff.**									
	2	8	-322.35	660.90	0.80	0.12	660.70			**significant diff.**								0.13	0.16
	4	10	-320.78	661.80	1.70	0.08	661.60	0.12	0.56	**significant diff.**						0.52	0.10	0.20	0.05
	5	13	-317.73	661.90	1.60	0.07	661.50			not significant		-0.15	0.49	not significant		1.15	0.30	0.20	0.06
	4	10	-320.87	662.00	1.80	0.07	661.70			**significant diff.**		-0.08	0.69			0.56	0.08	0.21	0.05
	5	13	-317.81	662.00	1.70	0.07	661.60	0.11	0.59	**significant diff.**				not significant		1.03	0.35	0.20	0.06
	2	8	-322.95	662.10	2.00	0.07	661.90			**significant diff.**						0.24	0.38		
	3	11	-319.88	662.10	1.90	0.07	661.80			**significant diff.**				not significant		0.76	0.48		

	**k**	**df**	**logLik**	**AICc**	**ΔAICc**	**ω**	**AIC**	**factor**	**Estimate**	**Std. Error**	**z value**	***P*-value**	**Effect on parasite**
**best model**	3	9	-320.95	660.10	0.00	0.18	659.90	**intercept**	-4.02	0.98	-4.11	< 0.001	
								**Mm *vs*.Em**	-0.05	0.43	-0.11	1.00	
								**Rr *vs*. Em**	**2.36**	**0.38**	**6.25**	**<1e-04**	*R. rattus > E. myoxinus*
								**Mr *vs*. Em**	**2.36**	**0.38**	**6.25**	**<1e-04**	*M. ravelobensis > E. myoxinus*
								**Rr *vs*. Mm**	**2.40**	**0.31**	**7.67**	**<1e-04**	*R. rattus > M.. murinus*
								**Mr *vs*. Mm**	0.75	0.36	2.09	0.15	
								**Mr *vs*. Rr**	**-1.65**	**0.30**	**-5.59**	**<1e-04**	*R. rattus > M.. ravelobensis*
								**forest category**	0.52	0.31	1.67	0.09	
								**dist. edge**	0.21	0.11	1.95	0.05	

spirurid egg 1 Model D full model: ~ sex + species + density + edge percentage

								sex		species		density		edge percentage	
	**k**	**df**	**logLik**	**AICc**	**ΔAICc**	**ω**	**AIC**	**Estimate**	***P*-value**	**Estimate**	***P*-value**	**Estimate**	***P*-value**	**Estimate**	***P*-value**
**models with ΔAICc < 2**	2	8	-193.64	403.60	0.00	0.53	403.30			**significant diff.**				-1.06	0.14
	1	7	-194.79	403.80	0.30	0.47	403.60			**significant diff.**					

	**k**	**df**	**logLik**	**AICc**	**ΔAICc**	**ω**	**AIC**	**factor**	**Estimate**	**Std. Error**	**z value**	***P*-value**	**Effect on parasite**
**best model**	2	8	-193.64	403.60	0.00	0.53	403.30	**intercept**	-1.85	0.75	-2.46	0.01	
								**Mm *vs*.Em**	-0.40	0.49	-0.80	0.85	
								**Rr *vs*. Em**	**2.46**	**0.41**	**6.04**	**<0.001**	*R. rattus > E. myoxinus*
								**Mr *vs*. Em**	0.60	0.51	1.17	0.64	
								**Rr *vs*. Mm**	**2.85**	**0.38**	**7.54**	**<0.001**	*R. rattus > M.. murinus*
								**Mr *vs*. Mm**	0.99	0.49	2.04	0.17	
								**Mr *vs*. Rr**	**-1.86**	**0.41**	**-4.52**	**<0.001**	*R. rattus > M.. ravelobensis*
								**edgepercent**	-1.06	0.71	-1.50	0.14	

spirurid egg 1 Model E full model: ~ sex + species + body condition + forest size + vegetation clearance

								sex		species		body condition		forest size		vegetation clearance	
	**k**	**df**	**logLik**	**AICc**	**ΔAICc**	**ω**	**AIC**	**Estimate**	***P*-value**	**Estimate**	***P*-value**	**Estimate**	***P*-value**	**Estimate**	***P*-value**	**Estimate**	***P*-value**
**models with ΔAICc < 2**	3	9	-169.08	356.60	0.00	0.63	356.20			**significant diff.**		**1.33**	**<0.001**			**-0.55**	**<0.001**
	4	10	-168.56	357.70	1.06	0.37	357.10			**significant diff.**		**1.36**	**<0.001**	-0.13	0.31	**-0.55**	**<0.001**

	**k**	**df**	**logLik**	**AICc**	**ΔAICc**	**ω**	**AIC**	**factor**	**Estimate**	**Std. Error**	**z value**	***P*-value**	**Effect on parasite**
**best model**	3	9	-169.08	356.60	0.00	0.63	356.20	**intercept**	-3.68	0.67	-5.45	< 0.001	
								**Mm *vs*.Em**	-0.41	0.52	-0.79	0.86	
								**Rr *vs*. Em**	**2.33**	**0.44**	**5.27**	**<0.001**	*R. rattus > E. myoxinus*
								**Mr *vs*. Em**	0.72	0.53	1.36	0.52	*M. ravelobensis > E. myoxinus*
								**Rr *vs*. Mm**	**2.74**	**0.40**	**6.82**	**<0.001**	*R. rattus > M.. murinus*
								**Mr *vs*. Mm**	1.13	0.48	2.37	0.08	
								**Mr *vs*. Rr**	**-1.61**	**0.41**	**-3.94**	**<0.001**	*R. rattus > M.. ravelobensis*
								**body cond.**	**1.33**	**0.42**	**3.15**	**< 0.001**	more parasites in hosts with better body condition
								**vegetation clearance**	**-0.55**	**0.19**	**-2.95**	**< 0.001**	less parasites in hosts from habitats with low vegetation density

spirurid egg 1 Model F full model: ~ sex + species + distance to edge + forest maturation

								sex		species		dist. edge		forest maturation	
	**k**	**df**	**logLik**	**AICc**	**ΔAICc**	**ω**	**AIC**	**Estimate**	***P*-value**	**Estimate**	***P*-value**	**Estimate**	***P*-value**	**Estimate**	***P*-value**
**models with ΔAICc < 2**	1	7	-178.06	370.40	0.00	0.41	370.10			**significant diff.**					
	2	8	-177.59	371.50	1.13	0.23	372.20			**significant diff.**		0.16	0.33		
	2	8	-177.74	371.80	1.44	0.20	371.50			**significant diff.**				0.16	0.43
	2	8	-178.01	372.40	1.98	0.15	372.00	-0.09	0.75	**significant diff.**					

	**k**	**df**	**logLik**	**AICc**	**ΔAICc**	**ω**	**AIC**	**factor**	**Estimate**	**Std. Error**	**z value**	***P*-value**	**Effect on parasite**
**best model**	1	7	-178.06	370.40	0.00	0.41	370.10	**intercept**	-2.59	0.61	-4.22	<0.001	
								**Mm *vs*.Em**	-0.39	0.51	-0.77	0.86	
								**Rr *vs*. Em**	**2.30**	**0.43**	**5.35**	**<0.001**	*R. rattus > E. myoxinus*
								**Mr *vs*. Em**	0.70	0.52	1.36	0.52	
								**Rr *vs*. Mm**	**2.69**	**0.39**	**6.90**	**<0.001**	*R. rattus > M.. murinus*
								**Mr *vs*. Mm**	1.10	0.47	2.34	0.09	
								**Mr *vs*. Rr**	**-1.60**	**0.39**	**-4.06**	**<0.001**	*R. rattus > M.. ravelobensis*

GPSR Model A full model: ~ sex + species + body condition + host species*cont. vs. frag. + cont. vs. frag. + vegetation clearance

	k	df	logLik	AICc	ΔAICc	ω	AIC	sex Estimate	sex *P*-value	species Estimate	species *P*-value	body condition Estimate	body condition *P*-value	host*cont. vs. frag. Estimate	host*cont. vs. frag. *P*-value	forest category Estimate	forest category *P*-value	vegetation clearance Estimate	vegetation clearance *P*-value
**models with ΔAICc < 2**	5	14	-1118.01	2264.50	0.00	0.72	2264.00			**significant diff.**		**0.23**	**0.02**	not significant		0.18	0.44	**-0.13**	**<0.001**
	6	15	-1117.93	2266.40	1.90	0.28	2265.90	0.03	0.68	**significant diff.**		**0.23**	**0.02**	not significant		0.18	0.44	**-0.13**	**<0.001**

	k	df	logLik	AICc	ΔAICc	ω	AIC	factor	Estimate	Std. Error	z/t value	*P*-value	Effect on parasite
**best model**	5	14	-1118.01	2264.50	0.00	0.72	2264.00	**intercept**	-0.23	0.25	-0.94	0.35	
								**Mm *vs* .Em**	**1.29**	**0.26**	**4.86**	**< 0.001**	*M. murinus > E. myoxinus*
								**Rr *vs* . Em**	**0.70**	**0.25**	**2.74**	**0.03**	*R. rattus > E. myoxinus*
								**Mr *vs* . Em**	**1.31**	**0.20**	**6.41**	**< 0.001**	*M. ravelobensis > E. myoxinus*
								**Rr *vs* . Mm**	-0.59	0.24	-2.40	0.07	
								**Mr *vs* . Mm**	0.02	0.19	0.13	1.00	
								**Mr *vs* . Rr**	**0.61**	**0.18**	**3.48**	**<0.001**	*M. ravelobensis > R. rattus*
								**body cond.**	**0.23**	**0.10**	**2.25**	**0.02**	more parasites in hosts with worse better condition
								**categoryfrag:hostmur**	-0.55	0.30	-1.83	0.07	
								**categoryfrag:hostrat**	0.23	0.29	0.78	0.43	
								**categoryfrag:hostrav**	-0.42	0.26	-1.62	0.11	
								**forest category**	0.18	0.23	0.78	0.44	
								**vegetation clearance**	**-0.13**	**0.04**	**-3.63**	**<0.001**	less parasites in hosts from habitats with low vegetation density

GPSR Model B full model: ~ sex + species + body condition + host species*cont. vs. frag. + cont. vs. frag. + forest size + forest maturation

	k	df	logLik	AICc	ΔAICc	ω	AIC	sex Estimate	sex *P*-value	species Estimate	species *P*-value	body condition Estimate	body condition *P*-value	host*cont. vs. frag. Estimate	host*cont. vs. frag. *P*-value	forest category Estimate	forest category *P*-value	forest size Estimate	forest size *P*-value	forest maturation Estimate	forest maturation *P*-value
**models with ΔAICc < 2**	5	14	-1122.85	2274.20	0.00	0.45	2273.70			**significant diff.**		**0.21**	**0.04**	not significant		-0.32	0.33	-0.05	0.12		
	4	13	-1124.04	2274.50	0.31	0.38	2274.10			**significant diff.**		**0.20**	**0.04**	**significant diff.**		0.05	0.82				
	6	15	-1122.77	2276.10	1.92	0.17	2275.50	0.03	0.70	**significant diff.**		**0.21**	**0.04**	not significant		-0.33	0.32	-0.05	0.11		

	k	df	logLik	AICc	ΔAICc	ω	AIC	factor	Estimate	Std. Error	z/t value	*P*-value	Effect on parasite
**best model**	5	14	-1122.85	2274.20	0.00	0.45	2273.70	**intercept**	0.34	0.41	0.83	0.41	
								**Mm *vs* .Em**	**1.26**	**0.27**	**4.73**	**< 0.001**	*M. murinus > E. myoxinus*
								**Rr *vs* . Em**	**0.73**	**0.26**	**2.84**	**0.02**	*R. rattus > E. myoxinus*
								**Mr *vs* . Em**	**1.34**	**0.21**	**6.52**	**< 0.001**	*M. ravelobensis > E. myoxinus*
								**Rr *vs* . Mm**	-0.53	0.25	-2.17	0.12	
								**Mr *vs* . Mm**	0.08	0.20	0.41	0.98	
								**Mr *vs* . Rr**	**0.62**	**0.18**	**3.49**	**< 0.001**	*M. ravelobensis > R. rattus*
								**body cond.**	**0.21**	**0.10**	**2.10**	**0.04**	more parasites in hosts with better condition
								**categoryfrag:hostmur**	-0.56	0.30	-1.85	0.06	
								**categoryfrag:hostrat**	0.19	0.29	0.65	0.52	
								**categoryfrag:hostrav**	-0.44	0.26	-1.66	0.10	
								**forest category**	-0.32	0.33	-0.98	0.33	
								**forest size**	-0.05	0.03	-1.57	0.12	

GPSR Model C full model: ~ sex + species + density + species*cont. vs. frag. + cont. vs. frag. + distance to edge

								sex		species		density		host*cont. vs. frag.		forest category		dist. edge	
	**k**	**df**	**logLik**	**AICc**	**ΔAICc**	**ω**	**AIC**	**Estimate**	***P*-value**	**Estimate**	***P*-value**	**Estimate**	***P*-value**	**Estimate**	***P*-value**	**Estimate**	***P*-value**	**Estimate**	***P*-value**
**models with ΔAICc < 2**	4.00	13.00	-1207.74	2441.90	0.00	0.51	2441.50			**significant diff.**				not significant		0.18	0.39	**0.15**	**<0.001**
	5.00	14.00	-1207.29	2443.10	1.10	0.28	2442.60	0.06	0.34	**significant diff.**				not significant		0.18	0.41	**0.15**	**<0.001**
	5.00	14.00	-1207.59	2443.70	1.70	0.21	2443.20			**significant diff.**		0.04	0.57	not significant		0.15	0.50	**0.15**	**<0.001**

	**k**	**df**	**logLik**	**AICc**	**ΔAICc**	**ω**	**AIC**	**factor**	**Estimate**	**Std. Error**	**z/t value**	***P*-value**	**Effect on parasite**
**best model**	4.00	13.00	-1207.74	2441.90	0.00	0.51	2441.50	**intercept**	-0.65	0.28	-2.30	0.03	
								**Mm *vs* .Em**	**1.03**	**0.23**	**4.47**	**< 0.001**	*M. murinus > E. myoxinus*
								**Rr *vs* . Em**	**0.72**	**0.24**	**3.01**	**0.01**	*R. rattus > E. myoxinus*
								**Mr *vs* . Em**	**1.27**	**0.19**	**6.72**	**< 0.001**	*M. ravelobensis > E. myoxinus*
								**Rr *vs* . Mm**	-0.31	0.21	-1.46	0.45	
								**Mr *vs* . Mm**	0.24	0.16	1.51	0.42	
								**Mr *vs* . Rr**	**0.55**	**0.17**	**3.28**	**0.01**	*M. ravelobensis > R. rattus*
								**categoryfrag:hostmur**	-0.25	0.27	-0.95	0.34	
								**categoryfrag:hostrat**	0.27	0.27	0.99	0.32	
								**categoryfrag:hostrav**	-0.43	0.24	-1.80	0.07	
								**forest category**	0.18	0.22	0.85	0.39	
								**dist. Edge**	**0.15**	**0.03**	**4.84**	**<0.001**	hosts with higher distance of their capture place to the forest edge have more parasites

GPSR Model D full model: ~ sex + species + density + edge percentage

								sex		species		density		edge percentage	
	**k**	**df**	**logLik**	**AICc**	**ΔAICc**	**ω**	**AIC**	**Estimate**	***P*-value**	**Estimate**	***P*-value**	**Estimate**	***P*-value**	**Estimate**	***P*-value**
**models with ΔAICc < 2**	2.00	9.00	-624.34	1267.10	0.00	0.68	1266.70			**significant diff.**				**-0.88**	**<0.001**
	3.00	10.00	-624.06	1268.60	1.40	0.32	1268.10	0.06	0.46	**significant diff.**				**-0.87**	**<0.001**

	**k**	**df**	**logLik**	**AICc**	**ΔAICc**	**ω**	**AIC**	**factor**	**Estimate**	**Std. Error**	**z/t value**	***P*-value**	**Effect on parasite**
**best model**	2.00	9.00	-624.34	1267.10	0.00	0.68	1266.70	**intercept**	0.55	0.31	1.79	0.12	
								**Mm *vs* .Em**	**0.89**	**0.13**	**6.69**	**<1e-04**	*M. murinus > E. myoxinus*
								**Rr *vs* . Em**	**1.07**	**0.13**	**8.11**	**<1e-04**	*R. rattus > E. myoxinus*
								**Mr *vs* . Em**	**1.01**	**0.16**	**6.39**	**<1e-04**	*M. ravelobensis > E. myoxinus*
								**Rr *vs* . Mm**	0.18	0.11	1.64	0.35	
								**Mr *vs* . Mm**	0.12	0.13	0.90	0.80	
								**Mr *vs* . Rr**	-0.06	0.14	-0.41	0.98	
								**edgepercent**	**-0.88**	**0.24**	**-3.65**	**<0.001**	more parasites in hosts from forest fragments with a lower percentage of edge habitat

GPSR Model E full model: ~ sex + species + body condition + forest size + vegetation clearance

	k	df	logLik	AICc	ΔAICc	ω	AIC	sex Estimate	sex *P*-value	species Estimate	species *P*-value	body condition Estimate	body condition *P*-value	forest size Estimate	forest size *P*-value	vegetation clearance Estimate	vegetation clearance *P*-value
models with ΔAICc < 2	3	10	-567.18	1154.90	0.00	0.72	1154.40			**significant diff.**		**0.41**	**<0.001**			**-0.13**	**0.01**
	4	11	-567.08	1156.80	1.92	0.28	1156.20			**significant diff.**		**0.41**	**<0.001**	0.02	0.67	**-0.12**	**0.02**

	k	df	logLik	AICc	ΔAICc	ω	AIC	factor	Estimate	Std. Error	z/t value	*P*-value	Effect on parasite
best model	3	10	-567.18	1154.90	0.00	0.72	1154.40	**intercept**	-0.38	0.27	-1.42	0.21	
								**Mm *vs*.Em**	**0.81**	**0.14**	**5.76**	**<0.001**	*M. murinus > E. myoxinus*
								**Rr *vs*. Em**	**0.95**	**0.14**	**6.76**	**<0.001**	*R. rattus > E. myoxinus*
								**Mr *vs*. Em**	**1.08**	**0.16**	**6.66**	**<0.001**	*M. ravelobensis > E. myoxinus*
								**Rr *vs*. Mm**	0.13	0.11	1.18	0.64	
								**Mr *vs*. Mm**	0.27	0.13	2.06	0.17	
								**Mr *vs*. Rr**	0.13	0.14	0.95	0.78	
								**body cond.**	**0.41**	**0.14**	**3.00**	**<0.001**	more parasites in hosts with better condition
								**vegetation clearance**	**-0.13**	**0.05**	**-2.46**	**0.01**	less parasites in hosts from habitats with low vegetation density

GPSR Model F full model: ~ sex + species + distance to edge + forest maturation

	k	df	logLik	AICc	ΔAICc	ω	AIC	sex Estimate	sex *P*-value	species Estimate	species *P*-value	dist. edge Estimate	dist. edge *P*-value	forest maturation Estimate	forest maturation *P*-value
models with ΔAICc < 2	2	9	-568.19	1154.80	0.00	0.58	1154.40			**significant diff.**		**0.19**	**<0.001**		
	3	10	-567.45	1155.40	0.61	0.42	1154.90			**significant diff.**		**0.19**	**<0.001**	0.08	0.21

	k	df	logLik	AICc	ΔAICc	ω	AIC	factor	Estimate	Std. Error	z/t value	*P*-value	Effect on parasite
best model	2	9	-568.19	1154.80	0.00	0.58	1154.40	**intercept**	-0.79	0.35	-2.24	0.06	
								**Mm *vs*.Em**	**0.86**	**0.14**	**6.16**	**<1e-04**	*M. murinus > E. myoxinus*
								**Rr *vs*. Em**	**1.01**	**0.14**	**7.26**	**<1e-04**	*R. rattus > E. myoxinus*
								**Mr *vs*. Em**	**1.03**	**0.16**	**6.42**	**<1e-04**	*M. ravelobensis > E. myoxinus*
								**Rr *vs*. Mm**	0.15	0.11	1.32	0.55	
								**Mr *vs*. Mm**	0.17	0.13	1.31	0.55	
								**Mr *vs*. Rr**	0.02	0.14	0.16	1.00	
								**dist. edge**	**0.19**	**0.06**	**3.35**	**<0.001**	hosts with higher distance of their capture place to the forest edge have more parasites
